# The distribution of dietary choline intake and serum choline levels in Australian women during pregnancy and associated early life factors

**DOI:** 10.1007/s00394-023-03186-w

**Published:** 2023-06-28

**Authors:** Lada Staskova, Wolfgang Marx, Samantha L. Dawson, Martin O’Hely, Toby Mansell, Richard Saffery, David Burgner, Fiona Collier, Boris Novakovic, Peter Vuillermin, Catherine J. Field, Deborah Dewey, Anne-Louise Ponsonby, The BIS Investigator Group

**Affiliations:** 1 The Florey Institute of Neuroscience and Mental Health, The University of Melbourne, 30 Royal Parade, Parkville, VIC 3052 Australia; 2 Deakin University, IMPACT - the Institute for Mental and Physical Health and Clinical Translation, School of Medicine, Barwon Health, Geelong, VIC 3220 Australia; 3 Murdoch Children’s Research Institute, Royal Children’s Hospital, Parkville, VIC 3052 Australia; 4 Department of Paediatrics, University of Melbourne, Parkville, VIC 3010 Australia; 5 Barwon Health, Geelong, VIC 3220 Australia; 6 Department of Agriculture, Food and Nutritional Science, University of Alberta, 4-126C Li Ka Shing Centre for Research, Edmonton, AB T6G 2H5 Canada; 7 Department of Pediatrics, Cumming School of Medicine, Alberta Children’s Hospital, Calgary, AB T3B 6A8 Canada; 8 Department of Community Health Sciences, Cumming School of Medicine, University of Calgary, Calgary, AB T2N 4Z6 Canada; 9 Owerko Centre, Alberta Children’s Hospital Research Institute, University of Calgary, Calgary, AB T2N 1N4 Canada

**Keywords:** Choline, Diet, Early life factors, Genetics, Recommended intake, Pregnancy, Metabolomics

## Abstract

**Background:**

Maternal dietary choline has a central role in foetal brain development and may be associated with later cognitive function. However, many countries are reporting lower than recommended intake of choline during pregnancy.

**Methods:**

Dietary choline was estimated using food frequency questionnaires in pregnant women participating in population-derived birth cohort, the Barwon Infant Study (BIS). Dietary choline is reported as the sum of all choline-containing moieties. Serum total choline-containing compounds (choline-c), phosphatidylcholine and sphingomyelin were measured using nuclear magnetic resonance metabolomics in the third trimester. The main form of analysis was multivariable linear regression.

**Results:**

The mean daily dietary choline during pregnancy was 372 (standard deviation (SD) 104) mg/day. A total of 236 women (23%) had adequate choline intake (440 mg/day) based on the Australian and New Zealand guidelines, and 27 women (2.6%) took supplemental choline ($\ge$ 50 mg/dose) daily during pregnancy. The mean serum choline-c in pregnant women was 3.27 (SD 0.44) mmol/l. Ingested choline and serum choline-c were not correlated (*R*^2^) = − 0.005, *p = *0.880. Maternal age, maternal weight gain in pregnancy, and a pregnancy with more than one infant were associated with higher serum choline-c, whereas gestational diabetes and environmental tobacco smoke during preconception and pregnancy were associated with lower serum choline-c. Nutrients or dietary patterns were not associated with variation in serum choline-c.

**Conclusion:**

In this cohort, approximately one-quarter of women met daily choline recommendations during pregnancy. Future studies are needed to understand the potential impact of low dietary choline intake during pregnancy on infant cognition and metabolic intermediaries.

**Supplementary Information:**

The online version contains supplementary material available at 10.1007/s00394-023-03186-w.

## Introduction

Choline is an essential dietary micronutrient and precursor for several important metabolites, necessary for many diverse functions, including cellular maintenance, neurotransmitter synthesis, lipid transport, and DNA methylation [1–4]. Some of the common dietary sources of high choline include eggs, meat products, whole grains and legumes [5]. For pregnant women, the recommended adequate intake from 19 to 50 years is 440-450 mg/day; this increases to 550 mg/day during lactation [5, 6]. The upper dietary intake level for choline during pregnancy and lactation is 3500 mg/day [5, 6]. Clinical interventional studies with large sample size and long-term follow up are limited to fully assess prenatal choline requirements and pregnancy outcomes [7]. Therefore, there are calls for further studies to determine choline dose–response during pregnancy relative to the infant’s development and to develop more accurate dietary guidelines across populations [8, 9]. There is a paucity of studies in free-living infant cohorts with concurrent data on prenatal diet, choline-related gene variants and prenatal serum levels of choline-containing compounds [9–13].

Endogenous synthesis of choline is insufficient, especially during high choline-demand life stages such as pregnancy and lactation. Studies have reported inadequate choline intake in pregnancy ranging from 268 to 319 mg/day in Belgium, Canada, and the US [9–13]; indeed in one large study (*n = *12,153) less than 1% of pregnant women had adequate intake relative to existing guidelines [11].

Vitamin and micronutrient supplementation is common during pregnancy; however, choline is rarely included in prenatal supplements [14]. Maternal choline supplementation has been shown to improve offspring cognition in human studies, but results have been inconsistent [15]. A likely factor for such inconsistency is the overlooked maternal confounding factors that can affect foetal development [15]. For example, socioeconomic status, maternal and paternal education, maternal stress, and nicotine and alcohol use may affect micronutrient intake and levels in the blood [16]. Therefore, when investigating dietary choline intake and supplementation during pregnancy relating to health outcomes in observational studies, potential confounders must be considered.

Blood measures of specific micronutrients are generally considered a gold standard to detect deficiencies rather than using estimates from dietary questionnaires. Unlike other micronutrients such as folate or vitamin B12, there is currently no standardised method or choline reference levels in the blood during pregnancy, [17], and choline concentrations are not routinely measured during pregnancy care.

**Fig. 1:**
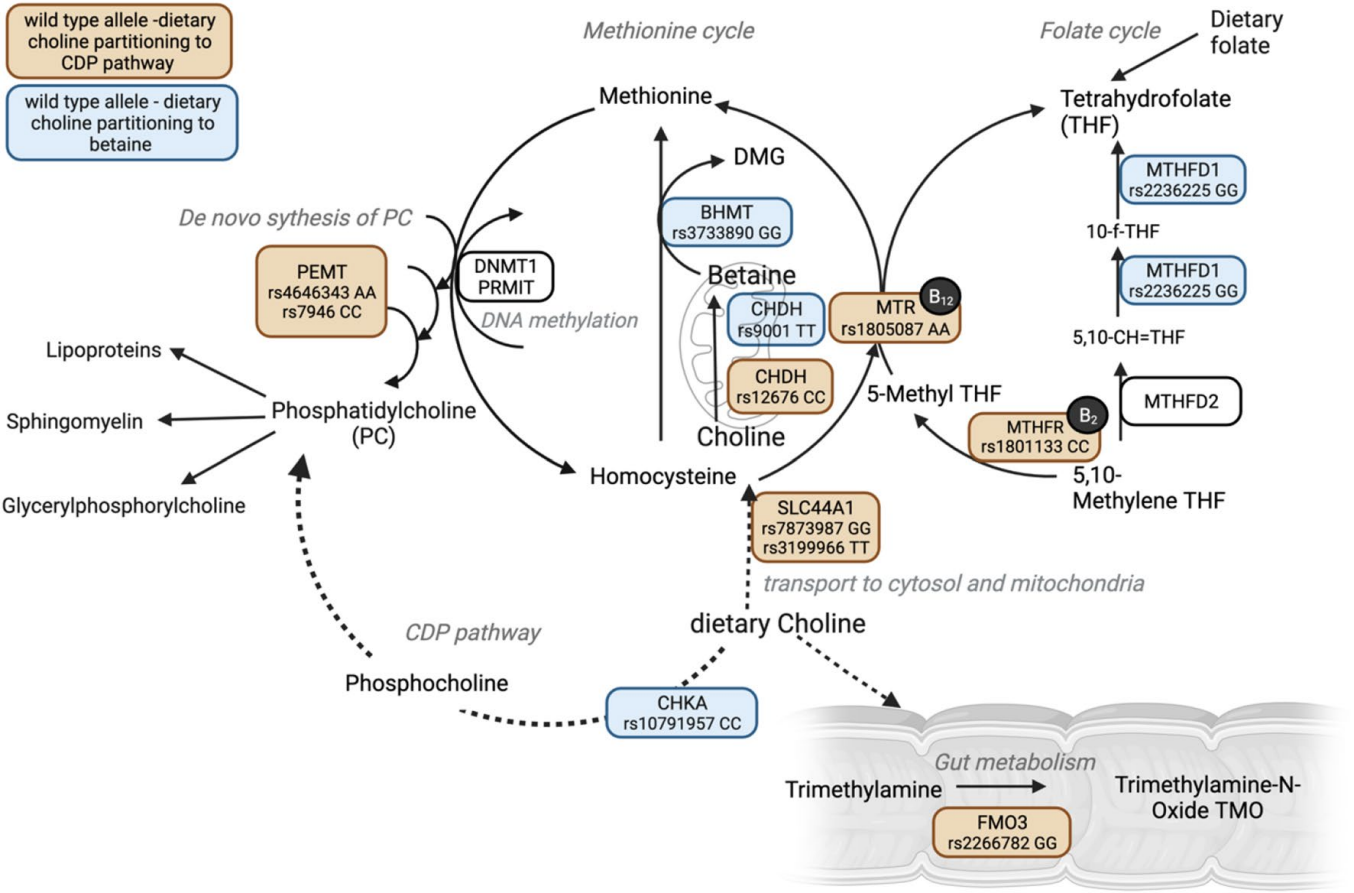
Schematic diagram of one-carbon cycle, (1C) choline metabolism and common genetic variants altering choline bioavailability (created in BioRender.com). SNPs partitioning dietary choline to CDP pathway in orange, SNPs partitioning dietary choline to betaine and therefore 1C cycle in blue. SNP ID and risk allele depicted for all 12 SNPs related to alter choline metabolism. *B12* vitamin B12; *B2*  vitamin B2; *CHDH* choline dehydrogenase; *BHMT* betaine-homocysteine *S*-methyltransferase; *MTR* methionine synthase; *DNMT1* DNA methyltransferase 1; *PEMT* phosphatidylethanolamine methyltransferase; *CDP* cytidine diphosphate; *CHKA* choline kinase alpha; *FMO3* flavin monooxygenase isoform 3; *MTHFR* 5,10-methylenetetrahydrofolate reductase; *MTHFD1/2* methylenetetrahydrofolate dehydrogenase; *DMG* dimethylglycine; *SLC44A1* solute carrier family 44 member 1; *PRMT* protein arginine methyltransferase

Endogenous choline synthesis occurs in the liver with production dependent on other nutrients such as folate, vitamin B12 and methionine [7, 18]. Dietary folate, choline and betaine (metabolite of choline [19]) are tightly linked due to the overlap in the one-carbon (1C) cycle metabolism (Fig. 1) [20]. The 1C cycle comprises of series of interlinking metabolic pathways essential for a range of biochemical processes including DNA, amino acid, and lipoprotein synthesis, as well as the generation of the universal one-carbon donor, S-Adenosylmethionine, critical for all methylation reactions including DNA methylation [21]. Choline-containing compounds can also be metabolised in the gut, which results in the production of trimethylamine, which is then absorbed and converted to trimethylamine-N-oxide (TMAO) in the liver [22]. Common genetic polymorphisms (SNPs) in genes involved in both choline-c and folate metabolism influence choline bioavailability [20]. However, research is generally lacking regarding the early life factors affecting serum choline-c levels as well as the effect of ingested choline on serum choline-c levels in pregnancy. Therefore, in this study we (i) describe the distribution of daily dietary intake, choline supplementation and serum choline-containing compound levels during pregnancy; (ii) document the extent to which dietary choline is associated with serum choline-c; and (iii) identify early life factors such as sociodemographic factors, family factors, prenatal factors, nutrients, diet and infant genetics that are associated with dietary and serum choline-c.

## Methods

### Study design and participants

From June 2010 to June 2013, a population-derived birth cohort (mothers, *n = *1064; infants, *n = *1074; 10 sets of twins) was recruited using an unselected antenatal sampling frame in the Barwon region of Victoria, Australia. Eligibility criteria, population characteristics and measurement details have been previously described [23]. From the inception cohort of *n = *1074, the present analysis was conducted in 948 pregnant women with available data on estimated prenatal dietary choline intake, maternal serum choline-c, and infant genetic data. The infant’s genotype information was used as a proxy for the mother as the latter was unavailable in this cohort. The BIS protocol was approved by the Barwon Health Human Research Ethics Committee (HREC 10/24) and the participating families provided written informed consent.

### Ingested choline intake estimation

The estimated dietary choline intake was calculated from the Dietary Questionnaire for Epidemiological Studies Version 2 (DQESv2) [24], obtained prenatally at 28 weeks of gestation. A total of 94 food items were used to calculate dietary choline intake. A comprehensive choline database developed and published previously [9] was used to estimate the choline content of specific foods. Food items in the BIS questionnaire were matched with the database and information on total dietary choline, choline-containing moieties (free choline, glycerophosphocholine, phosphocholine, phosphatidylcholine and sphingomyelin) and betaine were calculated based on the frequency of each food item that was reported during pregnancy. Food items were reported over the period of past 4 weeks from the date of completion. Dietary total choline is reported as the sum of all choline-containing moieties (free choline, glycerophosphocholine, phosphocholine, phosphatidylcholine and sphingomyelin). In this paper, we refer to the estimated daily dietary total choline intake as daily dietary choline intake. Betaine is reported separately.

The choline database [9] of > 2000 foods is based on the United States Department of Agriculture (USDA) Database for the Choline Content of Common Foods Release 2 [25]. Food items were classified into categories (e.g. dairy and eggs, baked products, or beef products) as outlined in the USDA database. Estimated daily dietary choline intake was adjusted for portion size using the Australian database published previously [26] to calculate choline intake in mg/day.

Choline supplementation was calculated and reported as daily supplementation during pregnancy if the reported dose was greater or equal to 50 mg/day. A total of six pregnant women took supplements that contained a dose of 20–35 mg; however, these were not consumed daily and were excluded. All choline supplementation was taken in the form of multivitamin or multimineral formulations. Choline supplementation was represented as a separate variable, total dietary choline intake represents choline intake from diet only.

### Metabolic measures

Non-fasting blood samples were collected by a Geelong pathology service in serum clotting tubes from pregnant women at approximately 28 weeks of gestation. Blood samples were centrifuged within 2–3 h of collection, and serum was removed and refrigerated before transport to the BIS laboratory for aliquoting and storage at − 80 °C. The Nightingale nuclear magnetic resonance-based metabolomics platform (Nightingale Health Ltd., Helsinki, Finland) was used to obtain concentrations for serum total choline-c (mmol/l), and choline-containing moieties including phosphatidylcholine (mmol/l), and sphingomyelin (mmol/l) [27]. Total choline-c was derived as a primary measure of the class containing all compounds with a choline head group.

### Potential lifestyle and environmental factors of choline status

Sociodemographic, family, and prenatal maternal factors were examined as possible factors associated with estimated dietary choline intake and serum choline-c levels. All factors were collected as part of a self-reported 1st and 2nd trimester questionnaire collected at 28 weeks of gestation. Fever during the third trimester was reported retrospectively (4 weeks after birth) using a self-reported questionnaire. Information on gestational diabetes was gathered through hospital record linkage collected between 24 and 28 weeks of gestation [28]. Sociodemographic factors included maternal and parental education, mother’s age at conception and socioeconomic factor that was calculated using the Index of Relative Socioeconomic Advantage and Disadvantage (IRSD) based on the residential address from the Census of Population and Housing: Socioeconomic Indexes For Area (SEIFA) in Australia 2016 [29]. Family factors included parity, birth order, number of children in the household at birth (0–10 years) and grandparents' ancestry. Prenatal maternal factors included pre-pregnancy BMI, maternal weight at 28 weeks of gestation, maternal weight gain during pregnancy (calculated as the difference between pre-pregnancy weight and maternal weight at 28 weeks of gestation), fever during trimester 3, gestational diabetes, maternal vitamin D (25-hydroxyvitamin D3 in nmol/l) as published previously [30], maternal smoking (any or none) and environmental tobacco smoke (ETS) during preconception or pregnancy (any or none), seasonal indicator (based on estimated UVR exposure at trimester 2 in standard erythemal doses) as described previously [31] and perceived stress as described previously [32].

### Potential prenatal nutritional factors

Dietary factors obtained from the 28-week food frequency questionnaire were assessed for their association with both daily dietary choline intake and serum levels. These included: energy intake, fibre intake, protein intake, iron intake, omega-3 and omega-6 fatty acid intake and supplementation, alcohol intake, fish oil supplementation, and folate intake and supplementation. Dietary patterns were derived using principal component analysis as previously described [33]. In summary, PC1 included a “Modern healthy dietary pattern” with high loadings of fish, nuts, eggs, green vegetables, and whole grains. PC2 was identified as a “Western dietary pattern” with high loadings on pasta chips, meat, take-away foods, and sweets. PC3 was identified as a traditional “Anglo-Australian” dietary pattern with high loadings on meat and vegetables. PC data were centered and scaled into *z*-score, where negative z-scores indicated intakes lower than the mean and positive *z*-scores indicated intakes above the mean for each pattern. Different diets such as Modern healthy dietary pattern, Western dietary pattern, Anglo-Australian dietary patterns as well as vegetarian diet and the Australian Recommended Food Score (ARFS) [34] based on adherence to the Australian Dietary Guidelines [35] were also included as a potential factor.

### Genetic profile

The whole-genome imputed SNP genotyping of infant samples was conducted using the Illumina GSA platform with methods reported previously [36]. Maternal genotype data are currently not available in BIS. In summary, genotype calling was performed by Erasmus MC University Medical Center with the GenTrain 3 algorithm in GenomeStudio and quality-control was performed in Plink 1.9. Genotype calling, quality control and imputation were all performed on the entire cohort as a single batch using cord blood and infant blood samples collected at birth and 12 months.

A total of 12 SNPs across nine genes, previously published [20, 37, 38] as common variants associated with altered choline metabolism were available in the BIS cohort. These SNPs were coded as follows: (i) risk allele (0) for dietary choline partitioning to CDP pathway (ii) risk allele (2) for dietary choline partitioning to betaine, and therefore, 1C cycle [37, 38]. The roles of these genes and SNPs in choline metabolism including dietary choline partitioning, are illustrated in Fig. 1, and relevant coding is summarised in Table S6. A pathway diagram was constructed using BioRender.com.

Misclassification analysis was conducted on the SNP data to account for using infant’s genotype information as a proxy for the mother’s. The latter was unavailable in the BIS cohort. Allele frequency was obtained from the BIS infants for each SNP and the probability of the maternal genotype was calculated given the infant’s genotype under the assumption of Hardy–Weinberg equilibrium. A worked example of the method is provided in Supplementary Data Box S1.

### Statistical methods

To assess dietary choline intake and serum choline-c distribution, summary statistics were used. To determine the correlation between dietary and serum choline-c Pearson correlations were used. To avoid type I error in multiple testing of the correlation between dietary choline intake and serum choline-c, a Bonferroni correction with *p < *0.001 limit was applied. For linear regressions, no adjustments for multiple comparisons were undertaken to reduce the risk of type II errors as previously outlined [39].

Unadjusted linear regression models were performed to estimate the associations between lifestyle, environment, nutritional factors and dietary choline intake. Multivariable linear regression models were used to estimate the association between lifestyle, environment, nutritional factors, genetic factors and serum choline-c, adjusted for gestational age at blood collection and infant’s sex, as previously reported [27] and time interval between maternal serum collection and storage as reported previously [40]. The goodness of fit is indicated by the relative contribution variables in each model.

Further detailed multivariable analysis was beyond the scope of this study. Serum choline-c measures were converted to µmol/l for the multivariable linear regression models. Data were analysed using STATA version 17 (StataCorp LP).

### Sensitivity analysis

To examine dietary choline density, rather than absolute levels, we also conducted a sensitivity analysis by adjusting for energy [41]. This sensitivity analysis was performed only on estimated daily dietary choline intake.

## Results

Dietary choline intake (total choline, free choline, glycerophosphocholine, phosphocholine, phosphatidylcholine and sphingomyelin) and serum choline-containing compounds (total choline-containing compounds, phosphatidylcholine and sphingomyelin) were normally distributed in the BIS cohort, therefore, no transformation was required. Participant characteristics are shown in Table 1. The average estimated daily dietary total choline intake was 372 mg/day (Table 2). A total of 236 (23%) women met the adequate intake of 440 mg/day during pregnancy. When examining choline supplementation, 27 (2.6%) women consumed choline supplementation daily during pregnancy (≥ 50 mg per dose). The average estimated daily dietary betaine intake was 442 mg/day. A positive correlation between dietary choline, dietary choline-containing moieties, and betaine (*p < *0.001 for all correlations) was observed (Table 3).

**Table 1:** Participant characteristics

Characteristics	*N = *948	Maternal sample	Inception cohort	
		Mean (SD), Median [IQR] or % (*n*)	*N = *1074	Mean (SD), Median [IQR] or % (*n*)
**Parent and Household factors**				
Mother's age at conception (years)	948	31.5 (4.7), 31.8 [28–34]	1074	31.3 (4.8), 31.7 [28–35]
Father's age at conception (years)	907	33.6 (5.7), 33.4 [30–37]	1024	33.5 (5.9), 33.3 [30–37]
Mother is university-educated	945	52.6% (497)	1068	51.3% (548)
Father is university-educated	929	36.2% (336)	1044	35.2% (367)
Socioeconomic index (lowest tertile)	937	32.2% (302)	1061	33.6% (357)
All grandparents are of European descent	940	74.6% (701)	1060	73.0% (774)
Child's sex	948		1074	
Female		48.3% (458)		48.3% (519)
**Prenatal factors**				
Pre-pregnancy BMI (kg/m^2^)	824	25.4 (5.4), 24.0 [22–28]	927	25.4 (5.5), 24.0 [22–28]
Maternal weight—28-week interview (kg)	764	80.9 (15.4), 78.1 [70–88]	866	80.9 (15.5), 78.1 [70–89]
Gestational age—blood collection (wks)	948	28.2 (1.2), 28.0 [28, 29]	1047	28.3 (1.3), 28.1 [28, 29]
Edinburgh Depression Scale risk category	682		766	
Low risk < 10		83.6% (570)		82.9% (635)
Moderate risk 10–12		10.9% (74)		11.0% (84)
High risk > 12		5.6% (38)		6.1% (47)
Any ETS during preconception or pregnancy	927	15.6% (145)	1049	16.9% (177)
Maternal smoking throughout pregnancy	940	5.2% (49)	1061	6.3% (67)
Gestational diabetes mellitus	812	4.9% (40)	908	4.8% (44)
Pre-eclampsia	915	3.0% (27)	1039	3.4% (35)
Any alcohol consumption during pregnancy	894	52.5% (469)	989	52.7% (521)
Vegetarian diet	944	0.6% (6)	1016	0.6% (6)
Folate supplementation during pregnancy	917	97.3% (892)	1030	97.4% (1,003)
Omega-3 supplementation in pregnancy	589	57.4% (338)	637	57.9% (369)
Omega-6 supplementation during pregnancy	919	0.2% (2)	1034	0.2% (2)
Fish oil supplementation during pregnancy	562	16.4% (92)	608	16.1% (98)

*BMI* body mass index; *ETS* environmental tobacco smoke; *T1 & T2* trimester 1&2

**Table 2:** Distribution of daily dietary total choline intake, dietary choline-containing moieties intake (free choline, phosphocholine, phosphatidylcholine, sphingomyelin, glycerophosphocholine), dietary betaine intake using the food frequency questionnaire and serum total choline-containing compounds and serum choline moieties (phosphatidylcholine, sphingomyelin) levels at 28 weeks of gestation

	Prenatal measures @ 28 weeks of gestation		
	Mean (SD)	Median [IQR]	*N*, %
Daily choline supplementation during pregnancy > 50 mg per dose			27, 2.6%
Meeting total choline adequate intake 440 mg/day			236, 23%
**Choline dietary intake** (*N = *1020)^a^			
Betaine (mg/d)	442 (158)	435 [325–548]	
Choline esters (mg choline/d)			
Free choline	102 (29)	100 [83–120]	
Glycerophosphocholine	61 (22)	56 [45–72]	
Phosphocholine	15 (5)	15 [12–18]	
Phosphatidylcholine	174 (59)	166.5 [133–208]	
Sphingomyelin	19 (7)	18 [14–23]	
Total choline	372 (104)	362 [303–434]	
Energy intake (kJ,d)	7428 (2344)	7182 [5811–8697]	
Choline per energy intake (mg/kJ)	0.052 (0.12)	0.05 [0.04–0.059]	
**Maternal blood** (*N = *1010)^b^			
Phosphatidylcholine (mmol/l)	2.87 (0.41)	2.85 [2.59–3.09]	
Sphingomyelin (mmol/l)	0.57 (0.09)	0.57 [0.51–0.63]	
Total choline-containing compounds (mmol/l)	3.27 (0.44)	3.25 [2.98–3.52]	

*AI* adequate intake used from the Nutrient Reference Values for Australia and New Zealand, including recommended dietary intakes published in 2006 [5]

^a^Mean for gestational age at food frequency questionnaire collectio*n = *28.1 weeks

^b^Mean for gestational age at serum collection, *n = *28.3 weeks

**Table 3:** Correlation among estimated daily dietary total choline intake, dietary choline moieties intake (free choline, phosphocholine, phosphatidylcholine, sphingomyelin, glycerophosphocholine), dietary betaine intake and serum total choline-containing compounds and serum choline moieties (phosphatidylcholine, sphingomyelin) at 28 weeks of gestation

	Diet							Supplementation	Serum		
Variables	TC	PC	SM	Betaine	Free choline	GPC	Pch	Choline bitartrate	TC	PC	SM
**Diet** (mg/day)											
Total choline	1.000										
Phosphatidylcholine	0.914***	1.000									
Sphingomyelin	0.839***	0.855***	1.000								
Betaine	0.415***	0.259***	0.121***	1.000							
Free choline	0.824***	0.592***	0.466***	0.669***	1.000						
Glycerophosphocholine	0.737***	0.462***	0.608***	0.276***	0.649***	1.000					
Phosphocholine	0.774***	0.523***	0.551***	0.297***	0.792***	0.798***	1.000				
**Supplementation** (mg/day)											
Choline bitartrate	−0.033	−0.040	− 0.068**	−0.001	0.018	− 0.053*	0.016	1.000			
**Serum** (mmol/l)											
Total choline-c	−0.005	−0.019	−0.048	0.037	0.025	0.004	0.032	0.030	1.000		
Phosphatidylcholine	−0.014	−0.026	−0.054*	0.026	0.013	− 0.001	0.021	0.024	0.985***	1.000	
Sphingomyelin	0.017	0.009	−0.014	0.051	0.035	0.005	0.044	0.029	0.821***	0.729***	1.000

*GPC* Glycerophosphocholine, *Pch* phosphocholine, *PC* phosphatidylcholine, *SM* sphingomyelin, *TC* total choline-containing compounds

***p < 0.01, **p < 0.05, *p < 0.1, Bonferroni correction for multiple testing *p* < 0.001 in bold

The average serum level for total choline-containing compounds was 3.27 mmol/l (SD 0.44) (Table 2). A positive correlation among serum measures of total choline-containing compounds and serum phosphatidylcholine and sphingomyelin (*p < *0.001) was observed. However, no correlation was observed between daily dietary choline intake and serum choline-c (*R*^2^ = − 0.005, *p = *0.880 for choline; Table 3). Choline supplementation was also not correlated with serum choline-c (*R*^2^ = 0.030, *p = *0.347). Data used in this analysis include duplicate prenatal measurements for pregnant women with twins (*N = *7). Analysis that removed twin measurements was also performed on all determining factors with no significant changes to the results observed.

### Commonly reported foods and nutritional factors contributing to dietary choline intake in the Barwon Infant Study

The top dietary food categories contributing most to higher estimated dietary choline intake were dairy and eggs with an average contribution of 22% (Table 4). The top two food items were eggs (9%) and flavoured milk, e.g. cocoa or hot chocolate (5%). The food category contributing the second most to higher dietary choline intake was baked products (12%), with grain bread (5%) and wholemeal (3%) being the top food items. Other food categories contributing to dietary choline intake were vegetables (10%), beef products (9%), chicken and turkey (8%) and fruit (7%). The food categories contributing most to higher dietary betaine intake are reported in Table S1, with 69% of betaine intake being from baked products such as multigrain and wholegrain bread. Dietary choline-containing moieties and their contribution to daily intake are reported in Table S2.

**Table 4:** Most commonly reported food categories contributing to total dietary choline intake in the Barwon Infant Study at 28 weeks of gestation

Rank	Total dietary choline contribution	%
	Food category	
1	Dairy and eggs	21.9
	Eggs	9
	Flavoured milk drink (cocoa, Milo)	5
2	Baked products	12.1
	Grain bread	5
	Wholemeal	3
3	Vegetables and vegetable products	10.3
	Potatoes roasted, fried, or cooked	3
	Broccoli	2
4	Beef product	8.8
5	Chicken and turkey	7.9
6	Fruit and fruit products	7.3
	Oranges and other citrus fruit	2
	Fruit juice	1
7	Cereal grains pasta and snacks	5.3
	Pasta, noodles	5.1
	Rice	0.1
8	Fish and shellfish product	4.3
9	Lamb	4.3
10	Pork products	3.4
11	Legume and legume products	2.9
	Soy milk	1
	Baked beans	0.8
13	Sugar and sweets	2.6
	Chocolate	1.6
	Ice cream	0.9
14	Breakfast cereal	2.1
	Weet bix	0.8
	Porridge	0.6
	Mixed dishes (meat pies, pasties, quiche)	2.0
15	Fast foods	1.4
16	Sausages and luncheon meats	1.1
17	Nut and seed products	0.9
18	Snacks	0.7
19	Spices and Herbs	0.6
20	Alcoholic beverages	0.1

Several nutritional factors were positively associated with greater daily dietary choline intake (Table 5). These included, energy intake (*β = *0.03 mg/day per kJ/day increase; 95% CI 0.03, 0.03; *p < *0.001), fibre intake (*β = *6.75 mg/day per g/day increase; 95% CI 6, 7.51; *p < *0.001), protein intake (*β = *2.9 mg/day per g/day increase; 95% CI 2.75, 3.05; *p < *0.001), iron intake (*β = *14.12 mg/day per g/day increase; 95% CI 12.97, 15.27; *p < *0.001), total dietary omega-3 (*β = *103.74 mg/day per g/day increase; 95% CI 95.3, 112.18; *p < *0.001), total dietary omega-6 (*β = *11.07 mg/day per g/day increase, 95% CI 9.73, 12.41; *p < *0.001) and folate intake (*β = *0.58 mg/day per µg/day increase; 95% CI 0.52, 0.64; *p < *0.001). Different diets were associated with higher daily dietary choline intake. For every increase in one SD in both the Modern healthy dietary pattern (PC1) and Western dietary pattern (PC2), daily dietary choline intake increased by 25.6 mg/day (95% CI 19.44, 31.7; *p < *0.001) and 60.55 mg/day (95% CI 55.37, 65.72; *p < *0.001) respectively. The food items with high positive loadings for the Modern healthy dietary pattern were fish, nuts, eggs, green vegetables, and wholegrains, whereas loadings for the Western dietary pattern were full-cream milk, pasta, chips, meat, and take-away foods, sweet biscuits, and confectionery products. PCA loadings are further detailed in the previously published study [33]. Positive loadings for food items reflect an increase in dietary choline in relation to a one SD increase in the dietary pattern, negative loading for food items reflect a decrease in dietary choline intake in relation to a one SD increase in the dietary pattern. A positive increase in daily dietary choline intake of 5.83 mg/day (95% CI 5.12, 6.54; *p < *0.001) was also observed for every unit increase in the Australian Recommended Food Score (ARFS).

**Table 5:** Factors associated with estimated mean change and relative contribution to daily dietary total choline intake and total choline-containing compounds levels at 28 weeks of gestation

	Daily total choline dietary Intake (mg/d)				Total choline-containing compounds in serum (µmol/l)^a^			
	*β*	95%CI	*p* value	Relative contribution^b^ (%)	*β*	95%CI	*p* value	Relative contribution^b^ (%)
**Sociodemographic**								
Mother's age at conception (years)	**− 2.09**	**− 3.44, − 0.74**	**0.002**	**0.8**	**9**	**3.27, 14.73**	**0.002**	**1.12**
Father's age at conception (years)	− 0.24	− 1.36, 0.89	0.678	− 0.08	2.16	− 2.66, 6.98	0.379	0.25
Median maternal and paternal education	5.27	− 0.98, 11.51	0.098	0.17	9.44	− 17.3, 36.17	0.489	0.24
SEIFA IRSD in lowest tertile	− 0.39	− 14.03, 13.25	0.955	− 0.1	12.42	− 46.27, 71.1	0.678	0.23
Mother is university-educated	7.41	− 5.39, 20.21	0.256	0.03	18.7	− 36.4, 73.8	0.506	0.21
Father is university-educated	− 4.57	− 18.1, 8.95	0.507	− 0.06	− 6.94	− 65.05, 51.17	0.815	0.3
**Family**								
All grandparents of North European descent	14.12	− 0.51, 28.74	0.058	0.26	6.94	− 55.38, 69.26	0.827	0.22
Parity above 1	− 1.43	− 14.31, 11.46	0.828	− 0.09	12.63	− 42.83, 68.09	0.655	0.19
Birth order				0.23				0.29
First	Reference				Reference			
Second	− 0.31	− 15.12, 14.5	0.967		25.36	− 38.81, 89.53	0.438	
Third	15.14	− 4.07, 34.35	0.122		− 37.63	− 120.35, 45.1	0.372	
Fourth or later	− 24.97	− 60.22, 10.28	0.165		111.11	− 40.26, 262.49	0.15	
Multiple Birth Indicator				− 0.09				0.99
None	Reference				Reference			
1st twin	− 26.75	− 103.88, 50.38	0.496		**429.62**	**79.25, 779.99**	**0.016**	
2nd twin	− 30.3	− 102.49, 41.89	0.41		**325.92**	**1.41, 650.42**	**0.049**	
Number of children in household at birth (0–10 years)				0.29				0.27
None	Reference				Reference			
One	6.46	− 8.09, 21	0.384		− 0.69	− 63.74, 62.35	0.983	
Two	**21.95**	**2.31, 41.59**	**0.029**		− 66.85	− 151.45, 17.76	0.121	
Three	− 20.41	− 75.48, 34.66	0.467		103.38	− 138.34, 345.1	0.401	
Four	111.04	− 92.11, 314.19	0.284		− 534.93	− 1393.88, 324.03	0.222	
Birth interval–BIS child and prior sibling (years)	6.46	− 8.09, 21	0.384	0.47	− 5.6	− 17.26, 6.05	0.346	− 0.09
**Prenatal**								
Pre-pregnancy BMI (kg/m^2^)	**− 1.37**	**− 2.57,− 0.17**	**0.025**	**0.45**	**− 6.82**	**− 12.24, − 1.39**	**0.014**	**1.01**
Maternal weight at 28-week interview (kg)	− 0.4	− 0.85, 0.04	0.074	0.27	− 1.32	− 3.36, 0.72	0.203	0.52
Maternal weight gain (kg)^c^	0.86	− 0.5, 2.21	0.216	0.07	**7.05**	**0.93, 13.17**	**0.024**	**0.95**
Fever 3rd trimester	**43.36**	**7.8, 78.92**	**0.017**	**0.53**	− 46.93	− 194.71, 100.84	0.533	0.19
Gestational diabetes mellitus^g^	29.15	− 3.07, 61.37	0.076	0.25	**− 186.63**	**− 332.95, − 40.31**	**0.012**	**1.07**
Folate (red cell) (nmol/l)	0.01	− 0.01, 0.03	0.17	0.13	0.01	− 0.07, 0.09	0.803	0.01
Perceived stress (pregnancy and 1st 6 months)	0.13	− 0.88, 1.13	0.802	− 0.09	− 1.8	− 6.11, 2.5	0.412	0.2
Seasonal indicator at trimester 2^d^	− 1.6	− 6.54, 3.33	0.524	− 0.06	− 16.56	− 37.73, 4.62	0.125	0.42
Maternal vitamin D (nmol/l)	0.25	− 0.2, 0.69	0.273	0.06	0.06	− 1.77, 1.89	0.947	− 0.19
Any maternal pregnancy smoking	− 8.39	− 26.41, 9.63	0.361	− 0.02	− 44.63	− 120.93, 31.68	0.251	0.33
Any ETS during preconception or pregnancy^g^	− 6.79	− 24.5, 10.93	0.452	− 0.04	**− 92.62**	**− 166.06, − 19.17**	**0.014**	**0.79**
**Nutrition and nutrients**								
Energy (kJ/day)	**0.03**	**0.03, 0.03**	** < 0.001**	**48.06**	0	− 0.01, 0.01	0.643	0.01
Fibre (g/day)	**6.75**	**6, 7.51**	** < 0.001**	**23.21**	1.82	− 1.99, 5.63	0.348	0.09
Protein (g/day)	**2.9**	**2.75, 3.05**	** < 0.001**	**58.96**	− 0.04	− 1.06, 0.98	0.94	− 0.01
Iron (g/day)	**14.12**	**12.97, 15.27**	** < 0.001**	**36.4**	0.73	− 5.64, 7.09	0.823	0
Total dietary omega-3 (g/day)	**103.74**	**95.3, 112.18**	** < 0.001**	**36.39**	24.67	− 21.6, 70.94	0.296	0.11
Omega-3 supplementation in preg. (yes vs no)	8.61	− 7.03, 24.25	0.28	0.03	29.8	− 41.75, 101.35	0.414	0.11
Total dietary omega-6 (g/day)	**11.07**	**9.73, 12.41**	** < 0.001**	**20.55**	5.48	− 1.17, 12.12	0.106	0.27
Alcohol (g/day)	4.74	− 0.03, 9.5	0.051	0.28	4.28	− 16.22, 24.78	0.682	0.01
Consistent fish oil supp. in preg. (yes vs no)	− 12.03	− 33.61, 9.54	0.274	0.03	23.62	− 77.78, 125.02	0.647	− 0.19
Folate (ug/day)	**0.58**	**0.52, 0.64**	** < 0.001**	**27.58**	0.02	− 0.28, 0.32	0.89	− 0.01
Folate supplementation in preg. (yes vs no)	− 5.6	− 46.84, 35.64	0.79	− 0.09	69.72	− 98.49, 237.94	0.416	0.24
**Dietary patterns**								
Modern healthy dietary pattern (PC1 z-score per 1 SD)^e^	**25.6**	**19.44, 31.77**	** < 0.001**	**6.05**	24.15	− 3.96, 52.26	0.092	0.3
Western dietary pattern (PC2 z-score per 1SD)^f^	**60.55**	**55.37, 65.72**	** < 0.001**	**34.17**	− 5.33	− 33.24, 22.58	0.708	0.01
Traditional Anglo-Australian diet (PC3 z-score per 1 SD)	3.11	− 3.26, 9.49	0.38	− 0.01	10.89	− 17.52, 39.29	0.452	0.05
ARFS score (per unit)	**5.83**	**5.12, 6.54**	** < 0.001**	**20.21**	− 0.25	− 3.76, 3.27	0.89	− 0.01
Vegetarian diet (yes vs no)	− 71	− 153.92, 11.91	0.093	0.18	− 193.78	− 546.34, 158.79	0.281	0.12
Choline supplementation in preg. (yes vs no)	− 33.25	− 73.05, 6.54	0.101	0.17	− 17.03	− 202.74, 168.69	0.857	0.22

β—mean change per unit increase in factor

*SEIFA* Socio-economic Indexes for Areas; *IRSD* Index of Relative Socioeconomic Disadvantage; *BMI* body mass index; *ETS* environmental tobacco smoke; *BIS* Barwon Infant Study; *ARFS score* Australian Recommended Food Score based on adherence to Australian Dietary Guidelines

^a^Serum total choline-containing compounds regression adjusted for gestational age at blood collection, child’s sex and time interval between maternal serum collection and storage

^b^R^2^ for the predictive model = 0.48 i.e. the model explains 48% of the variance in dietary choline intake

^c^Maternal weight gain during pregnancy calculated as the difference between pre-pregnancy weight and maternal weight gain at 28 weeks of gestation

^d^Estimated as UVR exposure in standard erythemal doses (trimester 1 and trimester 3 also not significant)

Omega-6 supplementation excluded due to low power *N = *2

^e^Modern healthy dietary pattern: high positive loadings on fish, nuts, eggs, green vegetables, and wholegrains; for every increase in one standard deviation of Modern healthy dietary pattern the mean increase of dietary total choline was 25 mg

^f^Western dietary pattern: high loadings of full-cream milk, pasta, chips, meat and take-away foods, sweet biscuits, and confectionery products

Detailed PCA loadings plot previously published^33^

^g^Gestational diabetes and any ETS during preconception and pregnancy were associated with higher maternal serum choline-containing compounds at 28 weeks of gestation, however, after adjusting for maternal weight at 28 weeks, these factors were no longer significant (*p = *0.056, *p = *0.066)

*p* < 0.05 in bold

### Lifestyle and environmental factors associated with maternal dietary choline intake

Lifestyle and environmental factors associated with absolute daily dietary choline intake are shown in Table 5. The only sociodemographic factor associated with daily dietary choline intake was maternal age at conception (*β = *− 2.09 mg/day per 1-year increase, 95% CI − 3.44, − 0.74; *p = *0.002). There was no evidence that other sociodemographic factors such as SEIFA IRSD, father’s age, maternal and paternal education were associated with daily dietary choline intake. A family factor associated with daily dietary choline intake was the number of children in household at birth, where a mean increase of 21.95 mg/day (95% CI 2.31, 41.59; *p = *0.029) was observed for women having two other older children in the household compared to no other children in the household. There was no evidence that other family factors such as parity, birth order, and birth interval were associated with higher daily dietary choline intake. Women who experienced fever during the third trimester of pregnancy on average had 43.36 mg/day (95% CI 7.8, 78.92; *p = *0.017) higher intake of choline compared to those not experiencing fever. For every 1 unit (kg/m^2^) increase in pre-pregnancy BMI, the mean decrease in daily dietary choline intake was 1.37 mg/day (95% CI − 2.57, − 0.17; *p = *0.025).

Factors associated with daily dietary choline-containing moieties (phosphatidylcholine and sphingomyelin) are shown in Table S3 and Table S4, respectively. Two family factors associated with daily dietary phosphatidylcholine intake included a mean increase of 11.06 mg/day (95% CI 0.17, 21.95; *p = *0.047) for those children being born third compared to being born first in the family order and a mean increase of 14.96 mg/day (95% CI 3.85, 26.08; *p = *0.008) for women having two older children in the household compared to no other children in the household. One prenatal factor associated with an increase in daily dietary phosphatidylcholine intake included, gestational diabetes (*β = *22.03 mg/day; 95% CI 3.8, 40.26; *p = *0.018). Nutrient factors such as energy, fibre, protein, and iron were associated with daily dietary phosphatidylcholine intake. Different diets such as Modern healthy dietary pattern (PC1) and Western dietary pattern (PC2) were associated with dietary phosphatidylcholine intake as well as vegetarian diet with a mean decrease of 75.89 mg/day (95% CI − 122.82, − 28.96; *p = *0.002) for women who were vegetarian compared to women who were not vegetarian during pregnancy. However, the number of women who were vegetarian during pregnancy was low (*N = *6). Three sociodemographic factors associated with a decreased daily dietary sphingomyelin intake were father’s age (*β = *− 0.08 mg/day per 1 year increase; 95% CI − 0.15, 0; *p = *0.037), median maternal and paternal education (*β = *− 0.44 mg/day per score increase; 95% CI − 0.85, − 0.02; *p = *0.039) and father’s university education (*β = *− 1.53 mg/day; 95% CI − 2.43, − 0.64; *p = *0.001).

### Dietary choline factors after adjusting for total energy

Energy intake explained 48% of the variation in dietary choline intake. Upon adjusting for energy intake, additional sociodemographic factors such as median maternal and paternal education (*β = *5.81 mg/day per score increase; 95% CI 1.31, 10.3; *p = *0.011) and maternal university education (*β = *9.78 mg/day per unit increase; 95% CI 0.6, 18.95; *p = *0.037) were positively associated with higher total dietary choline intake (Table S5). After adjusting for energy intake, an increase in total dietary choline intake was associated with gestation diabetes. Some prenatal factors such as perceived stress during pregnancy and in the 6 months post birth (*β = *− 0.92 mg/day per score increase, 95% CI − 1.64, − 0.2; *p = *0.012) and any maternal smoking (*β = *− 14.19 mg/day; 95% CI − 27.12, − 1.26; *p = *0.032) were associated with decreased total dietary choline intake. For dietary factors, only four factors (i.e. protein, total dietary, omega-3 fatty acid, and ARFS score) remained significant after adjusting for energy intake.

### Lifestyle and environmental factors associated with serum choline-c

Lifestyle and environmental factors associated with serum total choline-c are shown in Table 5. Older maternal age at conception was associated with higher serum total choline-c (*β = *9 µmol/l per 1 year increase; 95% CI 3.27, 14.73; *p = *0.002). One family factor was associated with serum total choline-c, having multiple births were associated with higher serum total choline-c. Family factors including grandparent ancestry, parity, number of children in household at birth (0–10 years), birth order and birth interval were not associated with serum total choline-c. Prenatal factors associated with lower serum total choline-c included higher pre-pregnancy BMI (*β = *− 6.82 µmol/l per one kg/m^2^ increase, 95% CI − 12.24, − 1.39; *p = *0.014), environmental tobacco smoke during preconception or pregnancy (*β = *− 92.62 µmol/l, 95% CI 166.06, − 19.17; *p = *0.014) and gestational diabetes (*β = *− 186.63 µmol/l; 95% CI − 332.95, − 40.31; *p = *0.012). Other prenatal factors such as perceived stress, red blood cell folate levels (at 28 weeks of gestation), and vitamin D did not show any association with serum total choline-c.

Factors associated with phosphatidylcholine and sphingomyelin serum levels are provided in supplementary documents (Table S3 and Table S4, respectively). For sphingomyelin serum levels, five prenatal factors were associated with lower levels which included, pre-pregnancy BMI (*β = *− 1.49 µmol/l per one kg/m^2^ increase, 95% CI − 2.64, − 0.33; *p = *0.012), gestational diabetes (*β = *-50.63 µmol/l, 95% CI − 81.56, − 19.7; *p = *0.001), season indicator at trimester 2 (*β = *− 6.46 µmol/l, 95% CI − 10.98, − 1.95; *p = *0.005), any maternal smoking (*β = *-20.52 µmol/l, 95% CI − 36.79, -4.26; *p = *0.013) and any environmental tobacco smoke during preconception and or pregnancy (*β = *− 25.87 µmol/l, 95% CI − 41.57, − 10.18; *p = *0.001). Nutritional factors such as omega-3 supplementation (*β = *21.76 µmol/l, 95% CI 6.37, 37.15; *p = *0.006) and Modern healthy dietary pattern (*β = *11.06 µmol/l, 95% CI 5.09, 17.03; *p < *0.001) were associated with higher serum sphingomyelin levels.

Further analysis was completed to explore maternal weight at 28 weeks of gestation as a possible confounder. After adjusting for maternal weight at 28 weeks, gestational diabetes and any ETS during preconception and pregnancy were no longer significantly associated with serum total choline-c and phosphatidylcholine. Adjusting for maternal weight at 28 weeks of gestation did not materially alter findings for sphingomyelin.

### The association between infant genetic variants and maternal serum total choline-c

A total of 12 infant SNPs previously identified to be associated with serum total choline-c were examined (Table 6). Two genes showed a moderately significant association with higher serum total choline-c. A mean increase in serum total choline-c of 108.1 µmol/l (95% CI 25.21, 190.98; *p = *0.011) was observed for infants with homozygous allele (CC) compared to the risk allele (AA) in the gene *PEMT* rs4646343. Likewise, an estimated mean increase in serum total choline-c of 80.12 µmol/l (95% CI 4.16, 156.08; *p = *0.039) was observed for infants with heterozygous allele (AC) compared to the risk allele (AA) at this locus. The gene *CHKA* rs10791957 involved in the phosphorylation of choline as the first step in the CDP-choline pathway also showed moderate evidence of an association. There was a mean increase of 67.93 µmol/l (95% CI 3.11, 132.75; *p = *0.04) in serum total choline-c for infants with heterozygous risk allele (AC) compared to the homozygous allele (AA), although for this locus the mean increase for the risk allele (CC) infants of 50.31 µmol/l (95% CI -32.18, 132.8; *p = *0.232) did not reach statistical significance. The contribution of the *PEMT* and the *CHKA* gene variation to serum total choline-c was < 1%.

**Table 6:** SNPs associated with serum total choline-containing compounds (µmol/l)

Total *N = *948	Allele frequency in BIS	SNPs related to choline demand^a^			Relative contribution (%)
		*β*	95%CI	*p* value	
**Methionine cycle in 1C pathway**					
BHMT gene rs3733890					0.21
0	8.3% (79)	Reference			
1	40.7% (386)	− 26.52	− 134.76, 81.73	0.631	
2	50.9% (483)	21.81	− 84.22, 127.85	0.686	
PEMT gene rs4646343^b^					**0.68**
0	19.0% (180)	Reference			
1	50.7% (481)	**80.12**	**4.16, 156.08**	**0.039**	
2	30.3% (287)	**108.1**	**25.21, 190.98**	**0.011**	
Additive model (0 to > 2)^c^		**49.07**	**8.88, 89.26**	**0.017**	**0.81**
PEMT gene rs7946					0.26
0	7.0% (66)	Reference			
1	35.7% (338)	− 18.86	− 135.53, 97.81	0.751	
2	57.4% (544)	33.44	− 79.54, 146.42	0.561	
CHDH rs9001					− 0.02
0	1.1% (10)	Reference			
1	15.7% (149)	− 25.2	− 306.87, 256.47	0.861	
2	83.2% (789)	0.06	− 273.97, 274.09	1	
CHDH rs12676					− 0.06
0	51.7% (490)	Reference			
1	39.7% (376)	3.01	− 57.47, 63.49	0.922	
2	8.6% (82)	− 4.37	− 110.25, 101.5	0.935	
**Folate cycle in 1C pathway**					
MTHFR gene rs1801133					0.22
0	46.5% (441)	Reference			
1	42.2% (400)	− 15.44	− 75.8, 44.91	0.616	
2	11.3% (107)	− 79.47	− 175.95, 17.01	0.106	
MTR gene rs1805087					− 0.03
0	65.4% (620)	Reference			
1	30.9% (293)	− 17.47	− 79.96, 45.02	0.583	
2	3.7% (35)	12.17	− 139.25, 163.59	0.875	
MTHFD1 rs2236225					− 0.05
0	18.2% (173)	Reference			
1	53.2% (504)	7.11	− 70.34, 84.57	0.857	
2	28.6% (271)	14.06	− 71.51, 99.62	0.747	
CDP (cytidine diphosphate-choline) pathway					
CHKA gene rs10791957					0.41
0	31.9% (302)	Reference			
1	49.2% (466)	**67.93**	**3.11, 132.75**	**0.04**	
2	19.0% (180)	50.31	− 32.18, 132.8	0.232	
Additive model (0 to > 2)^b^		24.64	− 15.19, 64.47	0.225	0.36
**Choline transport**					
SLC44A1 gene rs7873987					0.15
0	78.4% (743)	Reference			
1	19.9% (189)	− 43.75	− 115.94, 28.44	0.235	
2	1.7% (16)	− 86.38	− 303.33, 130.57	0.435	
SLC44A1 gene rs3199966					0.28
0	82.9% (786)	Reference			
1	16.1% (153)	− 67.2	− 144.99, 10.6	0.09	
2	0.9% (9)	− 81.15	− 368.76, 206.45	0.58	
**Microbial metabolism of trimethylamine in the gut**					
FMO3 rs2266782					0.06
0	32.7% (310)	Reference			
1	52.4% (497)	3.24	− 60.37, 66.85	0.92	
2	14.9% (141)	− 40.57	− 129.18, 48.05	0.369	

Relevant SNPs were also explored against dietary choline with no significance observed

*BIS* Barwon Infant Study; *1C* one-carbon cycle; *Genes*: *BHMT* betaine-homocysteine *S-*methyltransferase; *PEMT* phosphatidylethanolamine *N-*methyltransferase; *CHDH* choline dehydrogenase; *MTHFR* methylenetetrahydrofolate reductase; *MTR* methionine synthase; *MTHFD1* methylenetetrahydrofolate dehydrogenase 1; *CHKA* choline kinase alpha; *SLC44A1* solute carrier family 44 member 1; *FMO3* flavin monooxygenase isoform

^a^Serum total choline-containing compounds regression adjusted for gestational age at blood collection and child's sex and time interval between maternal serum collection and storage

Note: SNPs were coded as follows: (i) wild type allele (0) for dietary choline partitioning to CDP pathway (ii) wild type allele (2) for dietary choline partitioning to betaine

Interpretation of PEMT gene:

^b^*β = *93.52, estimated mean increase in serum choline-containing compounds is 93.52 µmol/l for children with heterozygous allele AC (1) compared to the homozygous reference allele AA (0) genotype

^b^*β = *111.4, estimated mean increase in serum choline-containing compounds is 111.4 µmol/l for children with homozygous allele CC (2) compared to the homozygous reference allele AA (0) genotype

^c^*β = *51.2, estimated mean increase in serum choline-containing compounds is 51.2 µmol/l for each C allele the child carries

*p* < 0.05 in bold

Misclassification analysis was performed to account for the use of the infant’s genotype instead of the mother’s, which is unavailable in the BIS cohort. The estimated mean increase in serum total choline-c was 98.15 µmol/l (95% CI 17.77, 178.52; *p = *0.017) for one unit increase in risk allele in the expected maternal rs4646343 risk score (Table S7). This can be compared to the original analysis using infant’s genotype where the estimated mean increase in serum total choline-c was 49.07 µmol/l (95% CI 8.88, 89.26; *p = *0.017) for each risk allele the infant carried (Table 6). When accounting for misclassification, the effect size and confidence intervals were consistently higher, but the *p* value and relative contribution remained unchanged. Thus, misclassification adjustment deattenuated the magnitude of association by accounting for measuring error when using infant’s genotypes instead of the mother’s genotype.

## Discussion

In this study, 23% of pregnant women met the recommended choline intake of 440 mg/day during pregnancy and only 27 (2.6%) of women were taking prenatal supplements containing choline ($\ge$ 50 mg per dose) daily during pregnancy. Similarly low dietary choline intake has been reported in many countries including Belgium, US, Canada and Australia [9–13].

The importance of choline supplementation during pregnancy is increasingly recognised. The American Academy of Paediatrics recognised choline as a ‘brain-building’ nutrient and called upon paediatricians to ensure pregnant women and young children have adequate intakes of choline [42]. In 2017, the American Medical Association (AMA) reported that prenatal choline supplementation is uncommon [43]. Similar to this study, a group in Canada reported that only 3% of pregnant women were supplementing choline during pregnancy [9]. Moreover, current prenatal multivitamins and supplements either do not contain choline at all or only very low amounts (30–50 mg), therefore adequate intake of choline during pregnancy is unlikely to be met via supplementation alone, in contrast to other nutrients such as folate and vitamin B 12 [14, 43]. Further, the importance of optimal choline intake during pregnancy is not commonly known; therefore, education and improved public health knowledge regarding the importance of choline during pregnancy, which highlights foods high in choline is warranted to provide adequate maternal dietary choline in the first place. Currently, the low intake of eggs, dairy products, meat, nuts, and legumes among Australian women in pregnancy likely results in low estimated dietary choline intake.

In addition, the adequate intake level for dietary choline was derived based on the intake required to prevent liver damage in healthy men and women, which was then adjusted for pregnancy and lactation based on choline accretion by the foetus and placenta and the amount secreted in human breast milk [5]. Therefore, there is a need to understand choline demands, particularly during pregnancy and explore whether the current choline recommendations are adequate for optimal foetal outcomes. Indeed, current literature suggests that doses above the recommended intake may be beneficial. Several clinical trials examined the effectiveness of choline in relation to cognition, visual and memory tasks, with many intervention doses ranging from 550 to 930 mg/day [44–46]. Greater daily choline intake was associated with increased blood concentrations of choline and betaine, increasing the methyl donor pool for DNA methylation that is essential to foetal development [45]. Choline supplementation also has an effect on other essential nutrients such as vitamin B-12 [47] and lysophosphatidylcholine enriched with DHA [48] status during pregnancy, supporting a functional relationship between these nutrients. Further clinical trial with higher dose intervention (930 mg/day vs 480 mg/day during third trimester) during pregnancy showed improved child memory at year seven [49].

In this study, we have outlined that both quantity and dietary patterns are associated with higher dietary total choline intake. Both the Modern healthy dietary pattern and Western dietary pattern were associated with higher dietary total choline intake. It is important to note that other dietary patterns (such as vegetarian or vegan dietary pattern) may impact choline intake therefore, in the future research, additional further dietary patterns should be examined in relation to low choline intake. Reduction in consumption of dairy and other animal products is increasingly common with plant-based diets increasing in popularity due to environmental and animal welfare concerns [50]. A global survey in 2019 reported that 40% of consumers are trying to reduce their consumption of animal proteins, while 10% are avoiding meat completely [50]. In addition, the plant-based milk alternatives have also reduced consumption of dairy milk [51]. Since the primary food sources of choline are milk, eggs and red meat, supplementation during pregnancy and lactation may be even more important as the intake of choline-rich food sources decline. Notably, a study in 2020 demonstrated no difference in water-soluble choline compounds in breastmilk between vegetarian lactating women and non-vegetarians [52]. Due to the low number of vegetarian pregnant women (*N = *6) in this study, we were unable to examine differences in breastmilk choline by vegetarian status.

The serum total choline-containing compounds at 28 weeks of pregnancy reported in this study were found to have a median of 3.25 mmol/l, which is slightly higher than that previously reported in pregnancy [53] (i.e. median of 2.62 mmol/l at 24–26 weeks of gestation). The mean for serum choline in a healthy adult population was previously reported to be approximately 11.7 µmol/l [54]. These findings indicate that serum levels are much higher during pregnancy. The concentration of total choline-containing compounds in maternal serum was not correlated with the estimated dietary intake of choline during the third trimester of pregnancy. It is of note that this study did not measure free choline from plasma but rather serum total choline-containing compounds. Further serum betaine measure was not available for analysis despite it being a choline-saving, methyl donor in the 1C cycle. Further, due to the nature of choline, a multi-functional nutrient involved in multiple pathways, multiple blood markers might be required to fully understand choline metabolism. For example, a study in 2009 suggested no association between moderate changes in the dietary intake of choline and blood status, possibly due to the interplay of choline and folate in the 1C cycle [55].

Here we investigated the role of genetic variants on choline metabolism. We used the infant genotype as a surrogate for the maternal genotype and demonstrated that this is a suitable proxy. The misclassification would likely be non-differential, biasing results towards the null yet gene variant- choline-containing compounds associations were evident, indicating that choline metabolism is dependent on enzymes related to the 1C cycle in particular PEMT, responsible for choline de novo synthesis [37, 38]. The methyl group required in the synthesis of phosphatidylcholine via the PEMT enzyme is partially from exogenous choline, betaine and possibly folate [37]. This is consistent with our finding of a link between genetic variation at *PEMT* with serum total choline-containing compounds. Further, studies have demonstrated that apart from low dietary choline resulting from low consumption of choline-rich foods, a polymorphism in the *PEMT* rs1235817 promoter region can result in reduced synthesis of choline and increase the odds of developing organ dysfunction [20]. Polymorphism in the *PEMT* gene has also been linked to preterm birth in a low choline environment [56, 57]. It has been suggested that the lack of agreement between dietary and serum total choline-containing compounds under conditions of folate adequacy may be due to the interaction of folate and methionine on choline levels [55, 58]. In the case of folate deficiency, the need for choline may be increased. For example, those that abuse alcohol become folate deficient; hence, more choline is required [59, 60]. Studies have found that polymorphism in the *MTHFR* gene is associated with reduced efficiency, requiring higher folate intake; therefore, supplementation may be crucial for pregnancies with these variants. Overall, it is important to explore 1C metabolites all at once and measure not only the key enzymes, but also genotypes of these as well as the intermediates in the pathway.

In this study, we examined possible lifestyle, environmental, and nutritional factors associated with dietary choline intake and serum total choline-containing compounds. To our knowledge, this is the first study to comprehensively investigate early life factors and their effect on total choline, phosphatidylcholine, and sphingomyelin in diet and serum.

### Key strengths and limitations

The key strength of this study is the comprehensive analysis of early life factors, including prenatal factors such as sociodemographic, family, maternal, dietary, and genetics factors. In addition, we undertook an extensive misclassification analysis to account for the use of the infant’s genotype. One limitation is that the frequencies of some genotypes in specific genes were low, possibly explaining the lack of association in other genes. A further limitation of this study is that serum choline-containing compounds were measured on non-fasted blood samples, and therefore, we were unable to measure levels on plasma free choline measure used to understand the levels of unbound, free choline available in the blood rather than bound total choline-containing compounds [61]. Therefore, the interpretation of bound total choline-containing compounds with respect to nutrition and dietary measures is to be taken with caution as these levels are influenced by lipoprotein metabolism [62, 63]. Nevertheless, an examination of phosphatidylcholine, and sphingomyelin with respect to nutritional choline intake is important as these choline-containing compounds have major biological roles [7].

We focussed on key choline elements, but other related choline factors are not available in this cohort such as trimethylamine N-oxide indicative of choline metabolism in the gut. Further, we acknowledge possible misclassification when deriving daily dietary choline intake measures from the food frequency questionnaire; however, as choline is in primary food groups such as dairy, eggs and meat, which are well captured in the validated questionnaire, the impact of any potential misclassification is reduced compared to other nutrients that are not as well characterised. Data used in this analysis were collected in 2010–2013 in Australia, therefore, choline reference values were used based on the Nutrient Reference Values for Australia and New Zealand [5]. The recommended intake during pregnancy is higher (450 mg) in countries such as Canada or the US [6], therefore, based on the National Institutes of Health (NIH) guidelines [64], a total of 207 women (20.3%) would have had adequate choline intake during pregnancy. Finally, BIS is a cohort that is relatively homogenous in terms of education level, socioeconomic index, and ethnicity and therefore, these results may not be generalisable across all populations.

### Future studies

Due to the close relationship among dietary choline, folate and other 1C nutrients, the role of choline bioavailability and epigenetic modification through 1C mechanisms should be further understood. Thus, understanding choline bioavailability, utilisation, and dependency and consequences on other nutrients such as folate and B12 are the next step away from a “one-size-fits-all” approach and towards personalised nutrition [65]. Overall, prenatal levels of all metabolites in the 1C cycle would ideally be measured at once rather than individually. Changes and interventions on an individual level, especially by specific healthy eating interventions during pregnancy, have enormous potential in improving public health. Further, there is evidence in the literature that the gut microbiome affects the utility and bioavailability of choline, therefore, in future studies, we suggest exploring the role of the gut on choline metabolome [65].

This is the first study exploring a comprehensive list of lifestyle and environmental factors in both dietary and serum choline-containing compounds. Our findings indicate that dietary choline intake was below the recommended ‘adequate’ level. Dietary choline intake did not correlate with serum choline-containing compounds, possibly due to choline being a multi-functional nutrient with other nutrients such as folate, and genetics of endogenous synthesis and the gut microbiome affecting choline bioavailability. Further, serum choline-containing compounds may also indicate plasma lipoprotein metabolism including hepatic very low-density lipoproteins (VLDL), rather than representing nutritional intake alone.

## Supplementary Information

Below is the link to the electronic supplementary material.

Supplementary file1 (DOCX 204 KB)

## Data Availability

Access to BIS data including all data used in this paper can be requested through the BIS Steering Committee by contacting the corresponding author. Requests to access cohort data are considered on scientific and ethical grounds and, if approved, provided under collaborative research agreements. Deidentified cohort data can be provided in Stata or CSV format. Additional project information, including cohort data description and access procedures, is available at the cohort study’s website http://www.barwoninfantstudy.org.au (accessed on 15 June 2023).
