# Supplementary material for: The distribution of dietary choline intake and serum choline levels in Australian women during pregnancy and associated early life factors
Source: Eur J Nutr. 2023 Jun 28;62(7):2855–72. doi: 10.1007/s00394-023-03186-w (PMC10468947; doi:10.1007/s00394-023-03186-w)
Supplement: Supplementary file 1 — Supplementary file1 (DOCX 204 KB) [file 394_2023_3186_MOESM1_ESM.docx]

**Supplemental Material**

**The distribution of dietary choline intake and serum choline levels in Australian women during pregnancy and associated early life factors**

**Table of Contents**

**Table S1** Most commonly reported food categories contributing to dietary betaine intake (mg/day) in the Barwon Infant Study at 28 weeks of gestation.

**Table S2** Most commonly reported food categories contributing to free choline, phosphocholine, phosphatidylcholine, glycerophosphocholine and sphingomyelin (mg/day) in the Barwon Infant Study at 28 weeks of gestation.

**Table S3** Factors associated with estimated mean change and relative contribution of dietary phosphatidylcholine intake and maternal phosphatidylcholine serum ( levels at 28 weeks of gestation.

**Table S4** Factors associated with estimated mean change and relative contribution dietary sphingomyelin intake and sphingomyelin serum at 28 weeks of gestation.

**Table S5** Factors associated with estimated mean change and relative contribution dietary total choline intake adjusted for energy (kJ/day) and dietary betaine intake unadjusted at 28 weeks of gestation

**Table S6** Coding of SNPs associated with serum total choline-containing compounds depending on dietary partitioning between the Cytidine diphosphate pathway (CDP), and betaine and therefore one carbon cycle

**Box S1** Methods used to incorporate child’s genotype for SNPs misclassification analysis

**Table S7** SNPs associated with serum total choline-containing compounds (µmol/l) after inferring mother’s genotype from the child

**Table S1** Most commonly reported food categories contributing to dietary betaine intake (mg/day) in the Barwon Infant Study at 28 weeks of gestation.

|  | Betaine | | |
| --- | --- | --- | --- |
| Rank | Food category (top two items) | % | |
| 1 | Baked products | 69 | |
|  | *Multigrain bread* | 29 | |
|  | *Wholegrain bread* | 18 | |
| 2 | Cereal grains, pastas and snacks | 10 | |
|  | *Pasta or noodles* | 10 | |
| 3 | Breakfast cereals | 10 | |
|  | *Weet Bix* | 5 | |
|  | *Sultana Bran, Fibre Plus, Bran flakes* | 3 | |
| 4 | Vegetables and vegetable products | 5 | |
|  | *Silverbeet or spinach* | 3 | |
| 5 | Mixed Dishes | | 2 |
| 6 | Fast foods | | 1 |
| 7 | Beef Product | | 1 |
| 8 | Finfish and shellfish products | | 1 |
| 9 | Dairy and Eggs | | 1 |
| 10 | Chicken and Turkey | | <1 |
| 11 | Lamb, veal and game | | <1 |
| 13 | Fruits and fruit products | | <1 |
| 14 | Snacks | | <1 |
| 15 | Sugars and Sweets | | <1 |
| 16 | Sausages and luncheon meats | | <1 |
| 17 | Beverages | | <1 |
| 16 | Nut and seed products | | <1 |
| 17 | Spices and Herbs | | <1 |
| 18 | Legumes and Legume Products | | <1 |

**Table S2** Most commonly reported food categories contributing to free choline, phosphocholine, phosphatidylcholine, glycerophosphocholine and sphingomyelin (mg/day) in the Barwon Infant Study at 28 weeks of gestation.

| Free Cho | | | Pch | | | PC | | | | SM | |  | GPC |  |
| --- | --- | --- | --- | --- | --- | --- | --- | --- | --- | --- | --- | --- | --- | --- |
| rank | Food category | % | Food category | % | Food category | | % | Food category | % | | Food category | % | Food category | % |
| 1 | Baked products | 27 | Dairy and eggs | 31 | Dairy and Eggs | | 21 | Chickens and Turkey | 28 | | Dairy and Eggs | 42 | Dairy and Eggs | 42 |
| 2 | Vegetables | 17 | Vegetables | 27 | Beef Products | | 16 | Dairy and Eggs | 28 | | Baked products | 15 | Baked products | 15 |
| 3 | Cereal grains, pastas, and snacks | 12 | Fruit | 10 | Chickens and Turkey | | 11 | Beef Products | 16 | | Fruits and fruit products | 8 | Fruit | 8 |
| 4 | Fruits | 11 | Chickens and turkey | 9 | Vegetables and vegetable products | | 8 | Lamb, veal and game | 5 | | Sugars and Sweets | 8 | Sugar and sweets | 8 |
| 5 | Dairy and Eggs | 10 | Legumes | 5 | Lamb, veal and game | | 6 | Pork products | 5 | | Vegetables and vegetable products | 6 | Vegetables | 6 |
| 6 | Legumes | 5 | Baked products | 4 | Finfish and shellfish products | | 6 | Finfish and shellfish products | 4 | | Finfish and shellfish products | 5 | Finfish and shellfish | 5 |
| 7 | Breakfast cereals | 3 | Sugar and sweets | 3 | Fruits and fruit products | | 5 | Mixed Dishes | 4 | | Pork products | 4 | Pork products | 4 |
| 8 | Chickens and Turkey | 2 | Lamb, veal and game | 2.4 | Pork products | | 5 | Sugars and Sweets | 3 | | Lamb, veal and game | 3 | Lamb, veal and game | 3 |
| 9 | Lamb, veal and game | 2 | Mixed dishes | 1.5 | Baked products | | 4 | Sausages and luncheon meats | 2 | | Beef Products | 2 | Beef products | 2 |
| 10 | Sugars and Sweets | 2 | Pork products | 1.5 | Cereal grains, pastas and snacks | | 4 | Fast foods | 2 | | Breakfast cereals | 2 | Breakfast cereals | 2 |
| 11 | Finfish and shellfish products | 2 | Finfish and shellfish products | 1 | Mixed Dishes | | 3 | Baked products | 1 | | Cereal grains, pastas and snacks | 2 | Cereal grains, pastas and snacks | 2 |
| 12 | Fast foods | 1 | Breakfast cereals | 1 | Legumes and Legume Products | | 3 | Fruits and fruit products | 0 | | Spices and Herbs | 1 | Spices and herbs | 1 |
| 13 | Spices and Herbs | 1 | Fast food | 1 | Breakfast cereals | | 2 | Vegetables and vegetable products | 0 | | Legumes and Legume Products | 1 | Legumes and legume products | 1 |
| 14 | Beef Products | 1 | Beef products | 1 | Sausages and luncheon meats | | 2 | Cereal grains, pastas and snacks | 0 | | Mixed Dishes | 1 | Mixed Dishes | 1 |
| 15 | Nut and seed products | 1 | Nut and seed products | 0.5 | Fast foods | | 2 | Legumes and Legume Products | 0 | | Fast foods | 1 | Fast Food | 1 |
| 16 | Pork products | 1 | Sausages and luncheon meats | 0.5 | Nut and seed products | | 1 | Breakfast cereals | 0 | | Chickens and Turkey | 1 | Chickens and Turkey | 1 |
| 17 | Mixed dishes | 1 | Spices and Herbs | 0 | Sugars and Sweets | | 1 | Nut and seed products | 0 | | Snacks | 1 | Snacks | 1 |
| 18 | Sausages and luncheon meats | 1 | Snacks | 0 | Snacks | | 1 | Snacks | 0 | | Sausages and luncheon meats | 0 | Sausages and luncheon meats | 0.5 |
| 19 | Snacks | 0 | Beverages | 0 | Spices and Herbs | | 0 | Spices and Herbs | 0 | | Nut and seed products | 0 | Nut and seed products | 0 |
| 20 | Beverages | 0 | Cereals grains and pastas | 0 | Beverages | | 0 | Beverages | 0 | | Beverages | 0 | Beverages | 0 |

Abbreviations: GPC diet, estimated dietary Glycerophosphocholine intake; Pch diet, estimated dietary phosphocholine intake; PC diet, estimated dietary phosphatidylcholine intake; SM diet, estimated diet sphingomyelin intake

**Table S3** Factors associated with estimated mean change and relative contribution of dietary phosphatidylcholine intake and phosphatidylcholine serum levels at 28 weeks of gestation.

|  | Phosphatidylcholine dietary Intake (mg/day) | | | | Phosphatidylcholine in serum (µmol/l) ^a^ | | | | | |
| --- | --- | --- | --- | --- | --- | --- | --- | --- | --- | --- |
|  | β | 95%CI | p-value | Relative Contribution^b^ (%) | | β | 95%CI | p-value | | Relative  Contribution^b^  (%) |
| **Sociodemographic** |  |  |  |  |  | |  |  | |  |
| Mother's age at conception (years) | -0.82 | -1.58,-0.05 | 0.038 | 0.33 | **7.73** | | **2.31,13.14** | | **0.005** | **0.93** |
| Father's age at conception (years) | 0.17 | -0.47,0.81 | 0.601 | -0.07 | 1.29 | | -3.26,5.85 | | 0.577 | 0.2 |
| Median maternal and paternal education | 2.93 | -0.62,6.47 | 0.105 | 0.16 | 4.2 | | -21.05,29.44 | | 0.744 | 0.18 |
| SEIFA IRSD in lowest tertile | -0.91 | -8.64,6.82 | 0.818 | -0.09 | 22.57 | | -32.82,77.96 | | 0.424 | 0.26 |
| Mother is university-educated | 4.99 | -2.27,12.26 | 0.178 | 0.08 | 6.08 | | -45.96,58.12 | | 0.819 | 0.14 |
| Father is university-educated | -3.53 | -11.22,4.16 | 0.368 | -0.02 | -9.53 | | -64.45,45.39 | | 0.734 | 0.28 |
| **Family** |  |  |  |  |  | |  | |  |  |
| All grandparents of North European descent | 6.78 | -1.54,15.1 | 0.11 | 6.78 | -6.76 | | -65.64,52.13 | | 0.822 | 0.18 |
| Parity above 1 | 6.78 | -1.54,15.1 | 0.11 | 6.78 | 3.68 | | -48.71,56.07 | | 0.89 | 0.14 |
| Birth order |  |  |  | 0.15 |  | |  | |  | 0.24 |
| First | Reference | |  |  | Reference | | | |  |  |
| Second | 2.45 | -5.95,10.85 | 0.567 |  | 11.65 | | -49.13,72.43 | | 0.707 |  |
| Third | **11.06** | **0.17,21.95** | **0.047** |  | -37.83 | | -116.19,40.54 | | 0.344 |  |
| Fourth or later | -4.02 | -24,15.97 | 0.694 |  | 111.35 | | -32.03,254.74 | | 0.128 |  |
| Multiple Birth Indicator |  |  |  | -0.2 |  | |  | |  | **1.06** |
| None | Reference | |  |  | Reference | | | |  |  |
| 1^st^ twin | -2.61 | -46.36,41.14 | 0.907 |  | **425.32** | | **93.65,756.99** | | **0.012** |  |
| 2^nd^ twin | -5.11 | -46.05,35.83 | 0.807 |  | **325.63** | | **18.44,632.82** | | **0.038** |  |
| Number of children in household at birth (0-10 years) |  |  |  | 0.52 |  | |  | |  | 0.33 |
| None | Reference | |  |  | Reference | | | |  |  |
| One | 5.28 | -2.95,13.52 | 0.208 |  | -11.02 | | -70.7,48.66 | | 0.717 |  |
| Two | **14.96** | **3.85,26.08** | **0.008** |  | -68.62 | | -148.72,11.47 | | 0.093 |  |
| Three | -8.57 | -39.75,22.61 | 0.59 |  | 105.25 | | -123.58,334.08 | | 0.367 |  |
| Four | 66.98 | -48.04,182 | 0.253 |  | -589.99 | | -1403.14,223.17 | | 0.155 |  |
| Birth interval – BIS child and prior sibling (years) | 5.28 | -2.95,13.52 | 0.208 | 0.18 | -4.28 | | -15.12,6.56 | | 0.438 | -0.02 |
| **Prenatal** |  |  |  |  |  | |  | |  |  |
| Pre-pregnancy BMI (kg/m2) | -0.39 | -1.08,0.3 | 0.265 | 0.03 | -4.96 | | -10.1,0.19 | | 0.059 | 0.69 |
| Maternal weight at 28-week interview (kg) | -0.08 | -0.33,0.17 | 0.538 | -0.08 | -0.77 | | -2.71,1.16 | | 0.434 | 0.33 |
| Maternal weight gain (kg)^c^ | 0.23 | -0.54,1.01 | 0.553 | -0.08 | **6.99** | | **1.19,12.79** | | **0.018** | **0.95** |
| Fever 3^rd^ trimester | 17.38 | -2.67,37.43 | 0.089 | 0.21 | -27.91 | | -168.38,112.56 | | 0.697 | 0.17 |
| Gestational diabetes mellitus^g^ | **22.03** | **3.8,40.26** | **0.018** | **0.53** | **-139.05** | | **-277.18,-0.91** | | **0.049** | **0.73** |
| Folate (Red Cell) (nmols/l) | 0 | -0.01,0.01 | 0.675 | -0.12 | 0 | | -0.08,0.07 | | 0.904 | -0.02 |
| Perceived Stress (pregnancy & 1^st^ 6 months) | 0.12 | -0.45,0.69 | 0.674 | -0.08 | -1.22 | | -5.29,2.85 | | 0.557 | 0.14 |
| Seasonal indicator at trimester 2^d^ | 0.43 | -2.37,3.23 | 0.764 | -0.09 | -11.65 | | -31.66,8.35 | | 0.253 | 0.28 |
| Maternal vitamin D (nmol/l) | 0.11 | -0.14,0.36 | 0.396 | -0.09 | -0.05 | | -1.76,1.67 | | 0.957 | -0.06 |
| Any maternal pregnancy smoking | -3.92 | -14.13,6.29 | 0.451 | -0.04 | -29.84 | | -101.93,42.26 | | 0.417 | 0.24 |
| Any ETS during preconception or pregnancy^g^ | -5.73 | -15.78,4.32 | 0.263 | 0.03 | **-73.42** | | **-142.75,-4.08** | | **0.038** | **0.58** |
| **Nutrition and Nutrients** |  |  |  |  |  | |  | |  |  |
| Energy (kJ/day) | **0.01** | **0.01,0.02** | **<0.001** | **35.23** | 0 | | -0.01,0.01 | | 0.708 | 0 |
| Fibre (g/day) | **3.01** | **2.55,3.46** | **<0.001** | **14.23** | 1.07 | | -2.53,4.68 | | 0.559 | 0.02 |
| Protein (g/day) | **1.56** | **1.46,1.65** | **<0.001** | **52.71** | -0.11 | | -1.08,0.86 | | 0.822 | -0.01 |
| Iron (g/day) | **6.88** | **6.18,7.58** | **<0.001** | **26.74** | -0.03 | | -6.06,6 | | 0.993 | -0.01 |
| Total dietary omega-3 (g/day) | **53.16** | **48.11,58.2** | **<0.001** | **29.59** | 17.59 | | -26.25,61.43 | | 0.431 | 0.05 |
| Omega-3 supplementation in preg. (yes vs no) | 8.71 | -0.24,17.66 | 0.056 | 0.43 | 13.6 | | -54.54,81.75 | | 0.695 | -0.09 |
| Total dietary omega-6 (g/day) | **5.07** | **4.28,5.87** | **<0.00** | **13.35** | 4.75 | | -1.54,11.05 | | 0.139 | 0.22 |
| Alcohol (g/day) | **3.41** | **0.71,6.12** | **0.013** | **0.5** | 2.26 | | -17.16,21.68 | | 0.819 | -0.01 |
| Consistent fish oil supp. in preg. (yes vs no) | -3.9 | -16.31,8.5 | 0.537 | -0.11 | 11.81 | | -84.76,108.38 | | 0.81 | -0.35 |
| Folate (ug/day) | **0.26** | **0.23,0.3** | **<0.001** | **17.15** | -0.01 | | -0.3,0.27 | | 0.919 | -0.01 |
| Folate supplementation in preg. (yes vs no) | 1.42 | -21.93,24.76 | 0.905 | -0.1 | 60.26 | | -98.79,219.31 | | 0.457 | 0.19 |
| **Dietary patterns** |  |  |  |  |  | |  | |  |  |
| Modern healthy dietary pattern (PC1 z-score per 1 SD)^e^ | **13.92** | **10.41,17.44** | **<0.001** | **5.54** | 12.79 | | -13.87,39.45 | | 0.347 | -0.11 |
| Western dietary pattern (PC2 z-score per 1SD)^f^ | **32.76** | **29.75,35.76** | **<0.001** | **30.99** | -2.46 | | -28.9,23.97 | | 0.855 | -0.21 |
| Traditional Anglo-Australian diet (PC3 z-score per 1 SD) | 2.88 | -0.74,6.5 | 0.118 | 0.14 | 6.6 | | -20.31,33.52 | | 0.63 | -0.19 |
| ARFS score (per unit) | **3.11** | **2.7,3.52** | **<0.001** | **17.79** | -0.84 | | -4.17,2.49 | | 0.621 | -0.19 |
| Vegetarian diet (yes vs no) | **-75.89** | **-122.82,-28.96** | **0.002** | **0.89** | -121.64 | | -455.72,212.44 | | 0.475 | -0.15 |
| Choline supplementation in preg. (yes vs no) | -18.09 | -40.61,4.43 | 0.115 | 0.15 | -32.46 | | -208,143.08 | | 0.717 | 0.18 |

β - mean change per unit increase in factor; p<0.05 in bold

^a^Serum phosphatidylcholine regression adjusted for gestational age at blood collection and child's sex and time interval between maternal serum collection and storage;

^b^R^2^ for the predictive model = 0.48 i.e., the model explains 48% of the variance in dietary choline intake;

^c^Maternal weight gain during pregnancy calculated as the difference between pre-pregnancy weight and maternal weight gain at 28 weeks of gestation;

^d^Estimated as UVR exposure in standard erythemal doses (trimester 1 and trimester 3 also not significant);

^e^Modern healthy dietary pattern: high positive loadings on fish, nuts, eggs, green vegetables, and wholegrains; for every increase in one standard deviation of modern healthy pattern the mean increase of dietary Phosphatidylcholine was 13.95mg.

^f^Western dietary pattern: high loadings on full-cream milk, pasta, chips, meat and take-away foods, sweet biscuits, and confectionery;

Detailed PCA loadings plot previously published^33^.

^g^Gestational diabetes and any ETS during preconception and pregnancy were associated with higher maternal serum phosphatidylcholine at 28 weeks of gestation, however, after adjusting for maternal weight at 28 weeks, these factors were no longer significant (p=0.123, p=0.13).

Omega-6 supplementation excluded due to low power N=2.

Abbreviations: SEIFA, Socio-Economic Indexes for Areas; IRSD, Index of Relative Socioeconomic Disadvantage; BMI, Body mass index; ETS, Environmental tobacco smoke; ARFS score, Australian Recommended Food Score based on adherence to Australian Dietary Guidelines.

**Table S4** Factors associated with estimated mean change and relative contribution fot dietary sphingomyelin intake and sphingomyelin serum at 28 weeks of gestation.

|  | Sphingomyelin dietary Intake (mg/day) | | | | Sphingomyelin serum (µmol/l)^a^ | | | | |  |
| --- | --- | --- | --- | --- | --- | --- | --- | --- | --- | --- |
|  | β | 95%CI | p-value | Relative Contribution^b^ (%) | | β | 95%CI | p-value | Relative Contribution^b^ (%) | |
| **Sociodemographic** |  |  |  |  |  | |  |  |  |  |
| Mother’s age at conception (years) | **-0.23** | **-0.32,-0.14** | **<0.001** | **2.3** | **1.73** | | **0.5,2.95** | **0.006** | **0.71** |  |
| Father’s age at conception (years) | **-0.08** | **-0.15,0** | **0.037** | **0.34** | 0.59 | | -0.44,1.62 | 0.263 | 0.03 |  |
| Median maternal and paternal education | **-0.44** | **-0.85,-0.02** | **0.039** | **0.32** | 4.19 | | -1.52,9.9 | 0.15 | 0.16 |  |
| SEIFA IRSD in lowest tertile | 0.67 | -0.23,1.58 | 0.143 | 0.11 | -0.85 | | -13.41,11.71 | 0.895 | -0.06 |  |
| Mother is university-educated | -0.81 | -1.66,0.03 | 0.06 | 0.25 | 9.46 | | -2.31,21.23 | 0.115 | 0.19 |  |
| Father is university-educated | **-1.53** | **-2.43,-0.64** | **0.001** | **1.03** | 1.14 | | -11.28,13.55 | 0.858 | -0.02 |  |
| **Family** |  |  |  |  |  | |  |  |  |  |
| All grandparents of North European descent | 0.21 | -0.77,1.18 | 0.675 | -0.08 | 12.78 | | -0.5,26.07 | 0.059 | 0.32 |  |
| Parity above 1 | -0.16 | -1.01,0.7 | 0.721 | -0.09 | 2.38 | | -9.47,14.23 | 0.694 | -0.04 |  |
| Birth order |  |  |  | 0.13 |  | |  |  | 0.26 |  |
| First | Reference | |  |  | Reference | | |  |  |  |
| Second | -0.24 | -1.22,0.74 | 0.629 |  | 8.76 | | -5,22.51 | 0.212 |  |  |
| Third | 0.92 | -0.35,2.19 | 0.157 |  | -10.76 | | -28.49,6.98 | 0.234 |  |  |
| Fourth or later | -1.16 | -3.49,1.17 | 0.327 |  | 13.92 | | -18.53,46.38 | 0.4 |  |  |
| Multiple Birth Indicator |  |  |  | -0.06 |  | |  |  | 0.65 |  |
| None | Reference | |  |  | Reference | | |  |  |  |
| 1^st^ twin | -2.11 | -7.2,2.99 | 0.417 |  | **81.86** | | **6.62,157.1** | **0.033** |  |  |
| 2^nd^ twin | -2.09 | -6.86,2.68 | 0.39 |  | 63.43 | | -6.25,133.11 | 0.074 |  |  |
| Number of children in household at birth (0-10 years) |  |  |  | 0.11 |  | |  |  | 0.1 |  |
| None | Reference | |  |  | Reference | | |  |  |  |
| One | 0.24 | -0.72,1.2 | 0.629 |  | 1.61 | | -11.92,15.13 | 0.816 |  |  |
| Two | 1.22 | -0.08,2.52 | 0.066 |  | -13.96 | | -32.12,4.19 | 0.131 |  |  |
| Three | -1.59 | -5.23,2.05 | 0.393 |  | 19.47 | | -32.39,71.33 | 0.461 |  |  |
| Four | 5.76 | -7.66,19.19 | 0.4 |  | -104.35 | | -288.64,79.93 | 0.267 |  |  |
| Birth interval – BIS child and prior sibling (years) | 0.24 | -0.72,1.2 | 0.629 | 0.06 | -1.76 | | -4.22,0.7 | 0.16 | -0.11 |  |
| **Prenatal** |  |  |  |  |  | |  |  |  |  |
| Pre-pregnancy BMI (kg/m2) | 0.05 | -0.03,0.13 | 0.25 | 0.04 | **-1.49** | | **-2.64,-0.33** | **0.012** | **0.69** |  |
| Maternal weight at 28-week interview (kg) | 0.02 | -0.01,0.05 | 0.237 | 0.05 | -0.35 | | -0.78,0.08 | 0.111 | 0.27 |  |
| Maternal weight gain (kg)^c^ | 0.03 | -0.06,0.12 | 0.495 | -0.07 | 0.76 | | -0.54,2.05 | 0.251 | 0.12 |  |
| Fever 3^rd^ trimester | 1.55 | -0.77,3.87 | 0.189 | 0.08 | -15.58 | | -47.28,16.11 | 0.335 | -0.02 |  |
| Gestational diabetes mellitus^g^ | 1.77 | -0.38,3.91 | 0.106 | 0.19 | **-50.63** | | **-81.56,-19.7** | **0.001** | **1.28** |  |
| Folate (Red Cell) (nmols/l) | 0 | 0,0 | 0.738 | -0.13 | 0 | | -0.01,0.02 | 0.646 | -0.07 |  |
| Perceived Stress (pregnancy & 1^st^ 6 months) | 0.03 | -0.03,0.1 | 0.311 | 0 | -0.63 | | -1.54,0.29 | 0.178 | 0.07 |  |
| Seasonal indicator at trimester 2^d^ | 0.2 | -0.13,0.53 | 0.233 | 0.04 | **-6.46** | | **-10.98,-1.95** | **0.005** | **0.74** |  |
| Maternal vitamin D (nmol/l) | 0 | -0.03,0.03 | 0.85 | -0.3 | -0.02 | | -0.42,0.38 | 0.922 | -1.1 |  |
| Any maternal pregnancy smoking | 0.89 | -0.3,2.08 | 0.144 | 0.11 | **-20.52** | | **-36.79,-4.26** | **0.013** | **0.58** |  |
| Any ETS during preconception or pregnancy | 0.68 | -0.49,1.86 | 0.254 | 0.03 | **-25.87** | | **-41.57,-10.18** | **0.001** | **1.04** |  |
| **Nutrition and Nutrients** |  |  |  |  |  | |  |  |  |  |
| Energy (kJ/day) | **0** | **0,0** | **<0.001** | **31.08** | 0 | | 0,0 | 0.464 | -0.09 |  |
| Fibre (g/day) | **0.17** | **0.11,0.22** | **<0.001** | **3.08** | 0.67 | | -0.14,1.49 | 0.105 | 0.13 |  |
| Protein (g/day) | **0.17** | **0.16,0.18** | **<0.001** | **47.17** | 0.05 | | -0.17,0.27 | 0.671 | -0.13 |  |
| Iron (g/day) | **0.62** | **0.53,0.7** | **<0.001** | **15.63** | 0.58 | | -0.78,1.94 | 0.399 | -0.08 |  |
| Total dietary omega-3 (g/day) | **5.27** | **4.64,5.89** | **<0.001** | **21.2** | 5.88 | | -4,15.76 | 0.243 | -0.01 |  |
| Omega-3 supplementation in preg. (yes vs no) | 0.23 | -0.81,1.28 | 0.664 | -0.13 | **21.76** | | **6.37,37.15** | **0.006** | **1.08** |  |
| Total dietary omega-6 (g/day) | **0.41** | **0.31,0.51** | **<0.001** | **6.31** | 1 | | -0.42,2.43 | 0.165 | 0.06 |  |
| Alcohol (g/day) | 0.16 | -0.15,0.48 | 0.312 | 0 | 0.79 | | -3.59,5.17 | 0.725 | -0.14 |  |
| Consistent fish oil supp. In preg. (yes vs no) | -0.74 | -2.18,0.71 | 0.316 | 0 | 18.51 | | -3.46,40.48 | 0.098 | 0.23 |  |
| Folate (ug/day) | **0.02** | **0.01,0.02** | **<0.001** | **6.4** | 0.02 | | -0.04,0.09 | 0.495 | -0.1 |  |
| Folate supplementation in preg. (yes vs no) | -1.31 | -4.04,1.42 | 0.347 | -0.01 | 23.7 | | -12.19,59.59 | 0.195 | 0.1 |  |
| **Dietary patterns** |  |  |  |  |  | |  |  |  |  |
| Modern healthy dietary pattern (PC1 z-score per 1 SD)^e^ | -0.27 | -0.69,0.16 | 0.218 | 0.05 | **11.06** | | **5.09,17.03** | **<0.001** | **1.25** |  |
| Western dietary pattern (PC2 z-score per 1SD)^f^ | **4.25** | **3.92,4.59** | **<0.001** | **38.23** | -2.31 | | -8.27,3.65 | 0.446 | -0.09 |  |
| Traditional Anglo-Australian diet (PC3 z-score per 1 SD) | -0.02 | -0.44,0.4 | 0.932 | -0.1 | 4.96 | | -1.1,11.03 | 0.109 | 0.13 |  |
| ARFS score (per unit) | **0.15** | **0.1,0.2** | **<0.001** | **3.03** | 0.27 | | -0.48,1.02 | 0.483 | -0.1 |  |
| Vegetarian diet (yes vs no) | **-13.44** | **-18.9,-7.99** | **<0.001** | **2.16** | -64.51 | | -139.77,10.76 | 0.093 | 0.15 |  |
| Choline supplementation in preg. (yes vs no) | **-2.91** | **-5.54,-0.27** | **0.031** | **0.37** | 10.41 | | -29.28,50.09 | 0.607 | -0.03 |  |

β – mean change per unit increase in factor; p<0.05 in bold

^a^Serum sphingomyelin regression adjusted for gestational age at blood collection and child’s sex and time interval between maternal serum collection and storage;

^b^R^2^ for the predictive model = 0.48 i.e., the model explains 48% of the variance in dietary choline intake;

^c^Maternal weight gain during pregnancy calculated as the difference between pre-pregnancy weight and maternal weight gain at 28 weeks of gestation;

^d^Estimated as UVR exposure in standard erythemal doses (trimester 1 and trimester 3 also not significant);

^e^Modern healthy dietary pattern: high positive loadings on fish, nuts, eggs, green vegetables, and wholegrains; for every increase in one standard deviation of modern healthy dietary pattern the mean increase of serum sphingomyelin was 11.06 µmol/l.

^f^Western dietary pattern: high loadings on full-cream milk, pasta, chips, meat and take-away foods, sweet biscuits and confectionery products;

Detailed PCA loadings plot previously published^33^).

^g^After adjusting for maternal weight at 28 weeks findings were not materially altered.

Omega-6 supplementation excluded due to low power N=2.

Abbreviations: SEIFA, Socio-Economic Indexes for Areas; IRSD, Index of Relative Socioeconomic Disadvantage; BMI, Body mass index; ETS, Environmental tobacco smoke; BIS, Barwon Infant Study; ARFS score, Australian Recommended Food Score based on adherence to Australian Dietary Guidelines.

**Table S5** Factors associated with estimated mean change and relative contribution dietary total choline intake adjusted for energy (kJ/day) and dietary betaine intake unadjusted at 28 weeks of gestation.

|  | Total dietary Choline adjusted (mg/day)^a^ | | | | Betaine dietary intake unadjusted (mg/day) | | | |
| --- | --- | --- | --- | --- | --- | --- | --- | --- |
|  | β | 95%CI | p-value | Additional Relative Contribution^b^ (%) | β | 95%CI | p-value | Relative Contribution^c^ (%) |
| **Sociodemographic** |  |  |  |  |  |  |  |  |
| Mother’s age at conception (years) | **-1.39** | **-2.36,-0.42** | **0.005** | 0.35 | **2.36** | **0.3,4.42** | **0.025** | **0.4** |
| Father’s age at conception (years) | -0.21 | -1.01,0.59 | 0.605 | 0.25 | 0.9 | -0.81,2.6 | 0.302 | 0.01 |
| Median maternal and paternal education | **5.81** | **1.31,10.3** | **0.011** | 0.13 | **27.79** | **18.42,37.16** | **<0.001** | **3.13** |
| SEIFA IRSD in lowest tertile | -1.37 | -11.18,8.45 | 0.785 | -0.39 | **-35.37** | **-56.11,-14.63** | **0.001** | **1** |
| Mother is university-educated | **9.78** | **0.6,18.95** | **0.037** | -0.04 | **44.06** | **24.74,63.38** | **<0.001** | **1.84** |
| Father is university-educated | -1 | -10.67,8.67 | 0.839 | 0.11 | **38.22** | **17.79,58.65** | **<0.001** | **1.23** |
| **Family** |  |  |  |  |  |  |  |  |
| All grandparents of North European descent | 7.12 | -3.4,17.64 | 0.185 | -0.11 | **37.47** | **15.37,59.57** | **0.001** | **0.99** |
| Parity above 1 | 7.12 | -3.4,17.64 | 0.185 | 0.02 | 9.31 | -10.29,28.91 | 0.352 | -0.01 |
| Birth order |  |  |  | -1.19 |  |  |  | -0.14 |
| First | Reference | |  |  | Reference | |  |  |
| Second | -2.2 | -12.95,8.54 | 0.687 |  | 12.35 | -10.41,35.1 | 0.287 |  |
| Third | 0.29 | -13.65,14.22 | 0.968 |  | 15.83 | -13.67,45.34 | 0.293 |  |
| Fourth or later | -11.98 | -37.5,13.53 | 0.357 |  | 3.19 | -50.96,57.33 | 0.908 |  |
| Multiple Birth Indicator |  |  |  | -1.18 |  |  |  | 0.39 |
| None | Reference |  |  |  | Reference | |  |  |
| 1^st^ twin | -0.6 | -56.32,55.13 | 0.983 |  | -93.45 | -211.42,24.53 | 0.12 |  |
| 2^nd^ twin | -11.96 | -64.1,40.18 | 0.653 |  | -103.07 | -213.49,7.34 | 0.067 |  |
| Number of children in household at birth (0-10 years) |  |  |  | -1.21 |  |  |  | 0.28 |
| None | Reference | |  |  | Reference | |  |  |
| One | -0.57 | -11.13,10 | 0.916 |  | **23.64** | **1.34,45.94** | **0.038** |  |
| Two | 4.44 | -9.82,18.7 | 0.542 |  | 28.27 | -1.84,58.38 | 0.066 |  |
| Three | -9.69 | -49.55,30.17 | 0.633 |  | 40.26 | -44.18,124.7 | 0.35 |  |
| Four | -67.64 | -215.16,79.87 | 0.368 |  | 111.91 | -199.57,423.39 | 0.481 |  |
| Birth interval – BIS child and prior sibling (years) | -1.2 | -3.04,0.64 | 0.2 | 1.35 | **-7.53** | **-11.69,-3.36** | **<0.001** | **1.96** |
| **Prenatal** |  |  |  |  |  |  |  |  |
| Pre-pregnancy BMI (kg/m2) | -0.11 | -0.99,0.77 | 0.799 | -1.46 | **-3** | **-4.85,-1.14** | **0.002** | **1.02** |
| Maternal weight at 28-week interview (kg) | -0.2 | -0.52,0.11 | 0.207 | -0.07 | -0.53 | -1.21,0.15 | 0.124 | 0.17 |
| Maternal weight gain (kg)^d^ | -0.93 | -1.91,0.06 | 0.064 | -0.36 | **3.48** | **1.42,5.53** | **0.001** | **1.22** |
| Fever 3^rd^ trimester | -7.72 | -33.35,17.91 | 0.555 | 0.49 | 42.77 | -11.75,97.3 | 0.124 | 0.15 |
| Gestational diabetes mellitus | **32.89** | **10.14,55.63** | **0.005** | 1.28 | 9.78 | -39.8,59.37 | 0.699 | -0.1 |
| Folate (Red Cell) (nmols/l) | 0 | -0.01,0.01 | 0.962 | -4.15 | 0 | -0.02,0.03 | 0.744 | -0.13 |
| Perceived Stress (pregnancy & 1^st^ 6 months) | **-0.92** | **-1.64,-0.2** | **0.012** | 0.13 | -0.09 | -1.62,1.44 | 0.909 | -0.1 |
| Seasonal indicator at trimester 2^e^ | -0.11 | -3.65,3.42 | 0.949 | -0.05 | 1.69 | -5.82,9.2 | 0.659 | -0.08 |
| Maternal vitamin D (nmol/l) | 0.04 | -0.29,0.37 | 0.82 | -3.82 | 0.48 | -0.17,1.12 | 0.146 | 0.35 |
| Any maternal pregnancy smoking | **-14.19** | **-27.12,-1.26** | **0.032** | -0.05 | **-48.67** | **-75.83,-21.52** | **<0.001** | **1.11** |
| Any ETS during preconception or pregnancy | -8.01 | -20.74,4.72 | 0.217 | 0.03 | **-39.09** | **-65.88,-12.31** | **0.004** | **0.72** |
| **Nutrition and Nutrients** |  |  |  |  |  |  |  |  |
| Energy (kJ/day) | **NA** | **NA** | **NA** | **NA** | **0.03** | **0.03,0.03** | **<0.001** | **20.14** |
| Fibre (g/day) | **-1.45** | **-2.41,-0.5** | **0.003** | **0.41** | **12.63** | **11.56,13.7** | **<0.001** | **34.6** |
| Protein (g/day) | **2.93** | **2.58,3.28** | **<0.001** | **10.86** | **2.25** | **1.92,2.57** | **<0.001** | **15.01** |
| Iron (g/day) | **2.24** | **0.4,4.08** | **0.017** | **0.**24 | **17.95** | **16.04,19.86** | **<0.001** | **24.99** |
| Total dietary omega-3 (g/day) | **18.53** | **5.27,31.79** | **0.006** | **0.**34 | **113.21** | **98.55,127.86** | **<0.001** | **18.39** |
| Omega-3 supplementation in preg. (yes vs no) | 5.58 | -6.3,17.46 | 0.357 | -5.69 | 21.01 | -4.27,46.28 | 0.103 | 0.27 |
| Total dietary omega-6 (g/day) | -1.41 | -2.92,0.1 | 0.067 | 0.12 | **16.67** | **14.61,18.73** | **<0.001** | **19.82** |
| Alcohol (g/day) | 3.12 | -0.31,6.56 | 0.075 | 0.11 | 3.6 | -3.71,10.92 | 0.334 | -0.01 |
| Consistent fish oil supp. In preg. (yes vs no) | -0.89 | -17.18,15.39 | 0.914 | -4.86 | 3.59 | -31.16,38.34 | 0.839 | -0.16 |
| Folate (ug/day) | 0 | -0.08,0.07 | 0.984 | -0.05 | **0.8** | **0.71,0.89** | **<0.001** | **22.17** |
| Folate supplementation in preg. (yes vs no) | -9.26 | -38.72,20.2 | 0.538 | 0.32 | 33.98 | -29.14,97.1 | 0.291 | 0.01 |
| **Dietary patterns** |  |  |  |  |  |  |  |  |
| Modern healthy dietary pattern (PC1 z-score per 1 SD)^f^ | 3.66 | -1.16,8.48 | 0.137 | 0.07 | **49.95** | **40.69,59.21** | **<0.001** | **9.87** |
| Western dietary pattern (PC2 z-score per 1SD)^g^ | -3.14 | -12,5.73 | 0.487 | -0.02 | **26.39** | **16.75,36.03** | **<0.001** | **2.67** |
| Traditional Anglo-Australian diet (PC3 z-score per 1 SD) | -4.33 | -8.94,0.28 | 0.066 | 0.13 | 5.29 | -4.48,15.05 | 0.288 | 0.01 |
| ARFS score (per unit) | **3.52** | **2.96,4.08** | **<0.001** | 6.75 | **7.65** | **6.53,8.78** | **<0.001** | **14.81** |
| Vegetarian diet (yes vs no) | -53.12 | -112.88,6.64 | 0.081 | 0.11 | 57.52 | -69.7,184.75 | 0.375 | -0.02 |
| Choline supplementation in preg. (yes vs no) | -9.78 | -38.27,18.71 | 0.501 | 0.08 | -6.52 | -67.19,54.14 | 0.833 | -0.1 |

^a^Total dietary choline regression adjusted for energy intake; p<0.05 in bold

β – mean change per unit increase in factor;

^b^Additional relative contribution = 10.86% of residual variance for protein intake, after adjusting for energy with relative contribution of 48.06%;

^c^R^2^ for the predictive model = 0.48 i.e., the model explains 48% of the variance in dietary choline intake;

^d^Maternal weight gain during pregnancy calculated as the difference between pre-pregnancy weight and maternal weight gain at 28 weeks of gestation;

^e^Estimated as UVR exposure in standard erythemal doses (1^st^ trimester and 3^rd^ trimester also not significant);

^f^Modern healthy dietary pattern: high positive loadings on fish, nuts, eggs, green vegetables, and wholegrains; for every increase in one standard deviation of modern healthy dietary pattern the mean increase of dietary total choline was 3.6mg after adjusting for energy.

^g^Western dietary pattern: high loadings on full-cream milk, pasta, chips, meat and take-away foods, sweet biscuits, and confectionery products;

Detailed PCA loadings plot previously published^33^).

Abbreviations: SEIFA, Socio-Economic Indexes for Areas; IRSD, Index of Relative Socioeconomic Disadvantage; BMI, Body mass index; ETS, Environmental tobacco smoke; BIS, Barwon Infant Study; ARFS score, Australian Recommended Food Score based on adherence to Australian Dietary Guidelines.

**Table S6** Coding of SNPs associated with serum total choline-containing compounds depending on dietary partitioning between the Cytidine diphosphate pathway (CDP) and betaine, and therefore one carbon cycle

| **Dietary choline partitioning to CDP pathway** | | |
| --- | --- | --- |
| SNP ID | Gene | Risk allele (0) |
| rs4646343 | *PEMT* | AA |
| rs7946 | *PEMT* | CC |
| rs12676 | *CHDH* | CC |
| rs1801133 | *MTHFR* | CC |
| rs1805087 | *MTR* | AA |
| rs7873987 | *SLC44A1* | GG |
| rs3199966 | *SLC44A1* | TT |
| rs2266782 | *FMO3* | GG |
| **Dietary choline partitioning to betaine** | | |
| SNP ID | Gene | Risk allele (2) |
| rs3733890 | *BHMT* | GG |
| rs9001 | *CHDH* | TT |
| rs2236225 | *MTHFD1* | GG |
| rs10791957 | *CHKA* | CC |

**Box S1 Methods used to incorporate child’s genotype for SNPs misclassification analysis.**

Worked example to take into account the use of the child’s genotype instead of the mother’s genotype which is unavailable in the Barwon Infant Study. The example below is used to demonstrate steps taken when performing the misclassification analysis.

*Example: BHMT gene*

1. Calculating allele frequencies

- frequency of allele A = freq. AA + (1/2) freq. AG = 0.083+(1/2*0.407) = 0.2865
- frequency of allele G = 1-freq A = 0.7125

1. Calculating probability of mother’s genotype given child’s genotype for all possible combinations (assuming the Hardy-Weinberg equilibrium)

|  | Probability of mother’s genotype given child’s genotype | | |
| --- | --- | --- | --- |
|  | Mother | | |
| Child | AA | AG | GG |
| AA | 0.2862135^a^ | 0.7117875 | 0 |
| AG | 0.14310675 | 0.4990005 | 0.35589375 |
| GG | 0 | 0.2862135 | 0.7117875 |

*^a^Example for:* P (mother is AA | Child is AA)


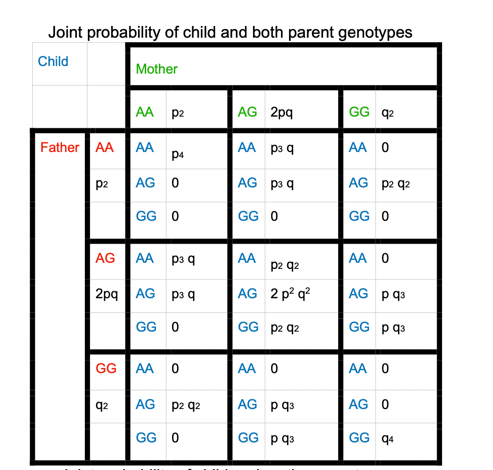
Step 1 to calculate joint probability of child and both parent genotypes P(child AA & mother AA)

P (child AA & mother AA & father AA) + P (child AA &mother AA& father AG) +

P (child AA & mother AA & father GG)

**🡪** = p^4^+p^3^q+0 = 0.023493

Step 2 to calculate P(child AA)

**🡪** p^2^ = 0.0820823

Step 3 to calculate P(mother is AA | child is AA)

**🡪** P(mother AA & child AA)/ P(child AA) = 0.28621

1. Calculate new scores using the probabilities and allocates scores based on the number of G alleles (scores reflect the dietary choline partitioning as outlined in method section)

|  | Calculating new score | | | New score | Original score |
| --- | --- | --- | --- | --- | --- |
|  | Mother | | |  |  |
| Child | AA=0 | AG=1 | GG=2 |  |  |
| AA | 0 | 0.7117875 | 0 | 0.7118 | 0 |
| AG | 0 | 0.4990005 | 0.7117875 | 1.2108 | 1 |
| GG | 0 | 0.2862135 | 1.423575 | 1.7098 | 2 |

1. Adjust new scores in regression and repeat for all 12 SNPs.

**Table S7** SNPs associated with serum total choline-containing compounds (µmol/l) after incorporating mother’s genotype from the child’s

|  | Total  N=948 | SNPs related to choline demand | | | Relative contribution (%) |
| --- | --- | --- | --- | --- | --- |
|  |  | β^a^ | 95%CI | p-value |  |
| **Methionine cycle in 1C pathway** | | | | | |
| BHMT gene rs3733890 |  | 54.58 | -32.08,141.24 | 0.217 | 0.37 |
| PEMT gene rs4646343 |  | **98.15** | **17.77,178.52** | **0.017** | **0.81** |
| PEMT gene rs7946 |  | 54.05 | -34.22,142.32 | 0.23 | 0.36 |
| CHDH rs9001 |  | 56.48 | -80.4,193.37 | 0.418 | 0.28 |
| CHDH rs12676 |  | 13.74 | -73.41,100.89 | 0.757 | 0.22 |
| **Folate cycle in 1C pathway** | | | | | |
| MTHFR gene rs1801133 |  | -57.65 | -140.53,25.22 | 0.173 | 0.41 |
| MTR gene rs1805087 |  | -18.74 | -119.22,81.73 | 0.714 | 0.22 |
| MTHFD1 rs2236225 |  | 27.19 | -54.74,109.12 | 0.515 | 0.25 |
| **CDP (cytidine diphosphate-choline) pathway** | | | | | |
| CHKA gene rs10791957 |  | 49.19 | -30.31,128.69 | 0.225 | 0.36 |
| **Choline transport** | | | | | |
| SLC44A1 gene rs7873987 |  | -75.2 | -197.15,46.75 | 0.227 | 0.36 |
| SLC44A1 gene rs3199966 |  | -97.01 | -234.96,40.94 | 0.168 | 0.41 |
| **Microbial metabolism of trimethylamine in the gut** | | | | | |
| FMO3 rs2266782 |  | -29.68 | -113.65,54.28 | 0.488 | 0.26 |

^a^Serum choline-containing compounds regression adjusted for gestational age at blood collection and child's sex time interval between maternal serum collection and storage;

*Interpretation of BHMT gene:* β = 47.87, estimated mean increase in serum choline containing compounds is 47.87µmol/l for one unit increase in the expected number of G alleles carried by the mother (one unit increase after misclassification reflect difference in child with AA (0.71) and AG (1.2108) genotype);

Relevant SNPs were also explored against dietary choline with no significance observed;

Abbreviations: Genes: BHMT, betaine homocysteine *S*-methyltransferase; PEMT, phosphatidylethanolamine *N-*methyltransferase; CHDH, choline dehydrogenase; MTHFR, methylenetetrahydrofolate reductase; MTR, methionine synthase; MTHFD1, methylenetetrahydrofolate dehydrogenase 1; CHKA, choline kinase alpha; SLC44A1, solute carrier family 44 member 1; FMO3, flavin monooxygenase isoform
